# Determinants of Brain Cell Metabolic Phenotypes and Energy Substrate Utilization Unraveled with a Modeling Approach

**DOI:** 10.1371/journal.pcbi.1002686

**Published:** 2012-09-13

**Authors:** Aitana Neves, Robert Costalat, Luc Pellerin

**Affiliations:** 1 Département de Physiologie, Université de Lausanne, Lausanne, Switzerland; 2 UMMISCO, UPMC Université Paris 6 and IRD, Paris, France; University of Virginia, United States of America

## Abstract

Although all brain cells bear in principle a comparable potential in terms of energetics, in reality they exhibit different metabolic profiles. The specific biochemical characteristics explaining such disparities and their relative importance are largely unknown. Using a modeling approach, we show that modifying the kinetic parameters of pyruvate dehydrogenase and mitochondrial NADH shuttling within a realistic interval can yield a striking switch in lactate flux direction. In this context, cells having essentially an oxidative profile exhibit pronounced extracellular lactate uptake and consumption. However, they can be turned into cells with prominent aerobic glycolysis by selectively reducing the aforementioned parameters. In the case of primarily oxidative cells, we also examined the role of glycolysis and lactate transport in providing pyruvate to mitochondria in order to sustain oxidative phosphorylation. The results show that changes in lactate transport capacity and extracellular lactate concentration within the range described experimentally can sustain enhanced oxidative metabolism upon activation. Such a demonstration provides key elements to understand why certain brain cell types constitutively adopt a particular metabolic profile and how specific features can be altered under different physiological and pathological conditions in order to face evolving energy demands.

## Author Summary

In an environment with appropriate oxygen levels (normoxia), most eukaryotic cells produce energy by oxidizing glucose into carbon dioxide and water. In this process, glucose is transformed into pyruvate, which then fuels oxidative phosphorylation in the mitochondria. Interestingly, Otto Warburg reported back in the 1920's that some eukaryotic cells prominently process glucose-derived pyruvate into lactate, hence “avoiding" the mitochondrial oxidation despite adequate oxygen concentrations. This phenomenon was termed *aerobic glycolysis* and was first observed in cancer cells. Since then, it has also been described in several normal tissues including the central nervous system. The biochemical basis of aerobic glycolysis has remained elusive until now. Taking advantage of a modeling approach, we unraveled the main metabolic characteristics that determine whether a cell will be strictly oxidative or rather will exhibit aerobic glycolysis. When applied in the context of the central nervous system, our findings not only provide a theoretical demonstration of why neurons and astrocytes differ in terms of metabolic profile, but also suggest that such complementarity forms the basis for metabolic cooperation between the two cell types.

## Introduction

A central question in biology concerns the biochemical characteristics that determine the metabolic profile (glycolytic vs. oxidative) of a particular cell type. Indeed, since the experimental description of aerobic glycolysis (i.e. the conversion of glucose into lactate despite the presence of sufficient oxygen levels to carry out oxidative metabolism) by Warburg [1], [2], this issue has become crucial to understand the process of tumorigenicity [3], [4], [5], [6]. Notwithstanding, numerous studies (including those of Warburg) have also documented the occurrence of aerobic glycolysis in several non-cancer cell types [7], [8], [9], [10]. Among normal tissues, the central nervous system was identified as partly relying on such a metabolic process for its energy production [11], [12]. At the cellular level, it was clearly demonstrated that astrocytes exhibit a prominent aerobic glycolytic activity [13]. In contrast, neurons seem devoid of this capacity [14] and rather present a strongly oxidative phenotype [15], [16], [17].

Such considerations acquire additional interest in the context of cell-cell interactions and metabolic cooperation. Indeed, in several tissues including muscles [18], testis [19], retina [20] as well as brain [21], a process known as intercellular lactate shuttle was reported that represents the supply of lactate produced by a glycolytic cell for the benefit of a neighboring oxidative cell [22]. Apart from the features that determine metabolic phenotypes, another crucial question is whether lactate provided by an adjacent cell can fulfill the energy requirements of the oxidative cell. For this purpose, we developed a modeling approach based on known biochemical characteristics of glucose and lactate metabolism. Our model allowed us to highlight key elements that are sufficient to explain the fundamental differences between an essentially oxidative cell and another cell exhibiting prominent aerobic glycolysis. Moreover, we could illustrate that under most physiological conditions, characteristics of lactate metabolism are adequate to sustain the evolving energy needs of the oxidative cell. Overall, the results of our modeling investigation account well for the described individual metabolic characteristics of astrocytes and neurons in the central nervous system, as well as for a prominent role of lactate as an energy substrate supplied by astrocytes to neurons, in accordance with the astrocyte-neuron lactate shuttle (ANLS) model.

## Models

### Description of the model

**Figure 1:**
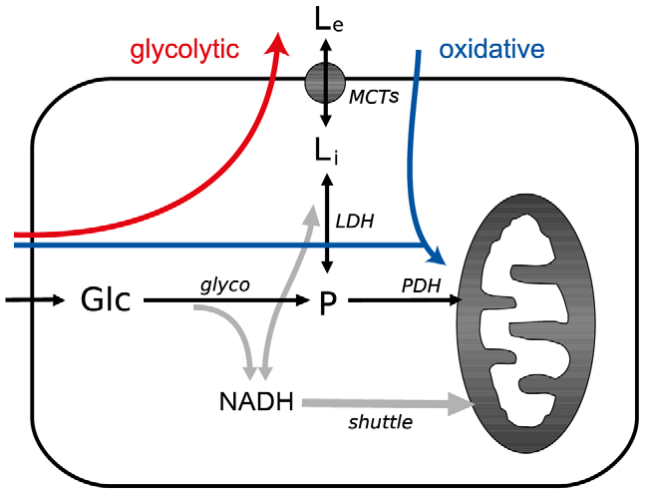
Model describing the simplified energetics of brain cells. Glucose undergoes glycolysis and all resulting pyruvate is either further metabolized by PDH or converted to lactate by LDH before being transported out of the cell. On the other hand, lactate can be transported into the cell and then metabolized into pyruvate by LDH. Because these processes require NAD^+^/NADH, we also modeled the “recycling" shuttle of NADH to NAD^+^ by mitochondria. The red arrow shows the metabolism of a typical predominantly glycolytic cell, characterized by lactate export; the blue arrow shows a typical oxidative phenotype, where both glucose and lactate import contribute to oxidative phosphorylation. Abbreviations: L_e_, extracellular lactate; L_i_, intracellular lactate; P, pyruvate; NADH, reduced nicotinamide-adenine dinucleotide; Glc, glucose; *J*_MCT_, transmembrane flux of lactate *via* MCTs; *J*_shuttle_, flux of NADH to NAD^+^ “recycling" by the mitochondria; *J*_glyco_, glycolytic flux; *J*_LDH_, metabolic flux *via* LDH; *J*_PDH_, metabolic flux *via* PDH.

In order to better understand the importance of distinct energy substrate sources for brain cell energetics, we considered a simple model taking into account glucose and lactate supply as well as their metabolism. This model describes the dynamics of lactate and glucose in brain cells. Fig. 1 shows a typical brain cell. Glucose (Glc) is metabolized into pyruvate (P) during glycolysis. This reaction requires NAD^+^, which is converted into NADH. For glycolysis to proceed, the excess NADH needs to be “recycled" back into NAD^+^. One way to do this is to convert pyruvate into lactate (L_i_), a reversible reaction catalyzed by lactate dehydrogenase (LDH). The produced lactate can then be transported outside the cell via the monocarboxylate transporters (MCT). The consumption of glucose for lactate production mainly is considered a glycolytic phenotype (red arrow in Fig. 1). Another alternative for the cell is to use the mitochondrial shuttles, mainly the malate-aspartate shuttle, to convert NADH into NAD^+^ in order to proceed with glycolysis. This can happen provided that the cell has enough mitochondrial activity (see Text S1 for more details). In this scenario, the pyruvate produced by glycolysis can be further metabolized by pyruvate dehydrogenase (PDH) into acetyl-CoA and produce energy via oxidative phosphorylation in the mitochondria. Since NADH is recycled into NAD^+^ by the mitochondrial shuttles, lactate can be imported into the cell and the LDH can perform the reverse reaction, converting it into pyruvate to feed oxidative phosphorylation as well. Such a phenotype is called oxidative (blue arrows in Fig. 1). As we will show, this model is very general and can be applied to both neurons and astrocytes, by modifying some biochemical conditions, namely the rate of NADH recycling to NAD^+^ (*J*_Shuttle_) and the rate of the PDH reaction (*J*_PDH_).

In this model, lactate is taken up (or released) by brain cells via MCTs and further metabolized by LDH into pyruvate (or produced *via* this reaction). The intracellular lactate dynamics is therefore described by the following equation: 
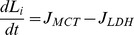
where 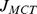 is the net transport of lactate inside the cell (i.e. if 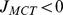, lactate is being transported *outside* the cell) and 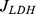 is the net rate of the reaction catalyzed by LDH; no *a priori* assumption is made about the sign of 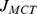 and 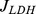.

In parallel to the LDH reaction, glucose uptake and glycolysis contribute to the balance of pyruvate, which is further metabolized by PDH into acetyl-CoA:
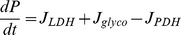
where 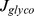 is the rate of pyruvate production by glycolysis and 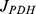 is the rate of pyruvate consumption by the reaction catalyzed by PDH. Then, acetyl-CoA is used to fuel the Krebs cycle and eventually oxidative phosphorylation in the mitochondria.

Another important feature of our model is the role of NADH. Indeed, both the forward LDH reaction and glycolysis produce NADH (from NAD^+^), while it is “recycled" back into NAD^+^ by the mitochondrial shuttles for the transfer of reducing equivalents:
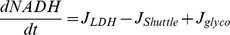
where 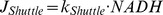 is the flux of mitochondrial NADH shuttling, that we assume proportional to NADH for simplicity (*k_Shuttle_* is a constant). The NADH mitochondrial shuttle therefore plays an essential role in determining the direction of the LDH reaction. (See Text S1 for a detailed description of the model).

### Role of lactate transport in fueling oxidative phosphorylation

We note that the baseline steady state values (subscript 0), obtained by equating to zero the right-hand side of all the differential equations, obey to
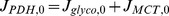


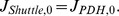
Let us define 
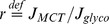
. Here we consider this ratio at basal state, namely 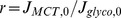. If we assume glycolysis to be constant, we have upon activation:
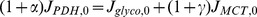
where α is the increase in acetyl-CoA production and γ the increase in transport flux, yielding: 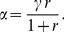
(1)Thus, given the ratio *r* at basal state, we can easily determine what will be the increase in oxidative phosphorylation upon transport activation (in the case where glycolysis is fixed).

### Choice of parameters

Some of the parameters in our equations, or a combination of them, have been measured or are known to lie within some range [17], [23], [24], [25], [26], [27]. The unknown parameters were set as to satisfy the following physiological constraints: the ratio 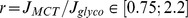 [15], [16], [28], [29], the intracellular lactate concentrations 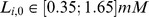 [30], [31], the ratio 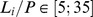 (chosen around the measured value 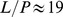 in 10-day old mouse brain [32] and considering that this ratio lies within [7]; [25] in human blood under various conditions [33]) and the redox state 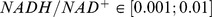 [34]. The parameters used to compute the basal state of a typical oxidative cell are listed in Table S1. The parameters used in all the other simulations are the same as for the oxidative cell (Table S1), except when mentioned (see the text and figure legends where the modified parameters are compared to the “reference" oxidative cell parameters).

## Results

All the figures presented in this work (except Fig. 1) were obtained by numerically solving in MATLAB® Eqs. (S1) in Text S1. For Table 1, we used Eq. (1) (see also Models).

**Table 1:** Importance of lactate transport regulation.

*r* \ γ	0.1	0.2	0.3	0.4	0.5	0.6	0.7	0.8
0.1	0.0091	0.018	0.027	0.036	0.046	0.055	0.064	0.073
0.4	0.029	0.057	0.086	*0.11*	*0.14*	*0.17*	*0.20*	*0.23*
0.7	0.041	0.082	*0.12*	*0.16*	*0.21*	*0.25*	*0.29*	**0.33**
1.0	0.050	*0.10*	*0.15*	*0.20*	*0.25*	**0.30**	**0.35**	**0.40**
1.3	0.057	*0.11*	*0.17*	*0.23*	*0.28*	**0.34**	**0.40**	**0.45**
1.6	0.062	*0.12*	*0.18*	*0.25*	**0.31**	**0.37**	**0.43**	**0.49**
1.9	0.066	*0.13*	*0.20*	*0.26*	**0.33**	**0.39**	**0.46**	**0.52**
2.2	0.069	*0.14*	*0.21*	*0.28*	**0.34**	**0.41**	**0.48**	**0.55**
2.5	0.071	*0.14*	*0.21*	*0.29*	**0.36**	**0.43**	**0.50**	**0.57**
2.8	0.074	*0.15*	*0.22*	*0.29*	**0.37**	**0.44**	**0.52**	**0.59**
3.1	0.076	*0.15*	*0.23*	**0.30**	**0.38**	**0.45**	**0.53**	**0.60**
3.4	0.077	*0.15*	*0.23*	**0.31**	**0.39**	**0.46**	**0.54**	**0.62**

Increases of oxidative metabolism, *J*_PDH_, obtained with distinct ratios of lactate/glycolysis-derived pyruvate, *r*, and different increases in lactate transport flux, γ (cf. Eq. (1)). Roman: below 10%; italic: between 10 and 29%; boldface: above 29%.

### Key parameters defining metabolic phenotypes (oxidative vs. glycolytic)

**Figure 2:**
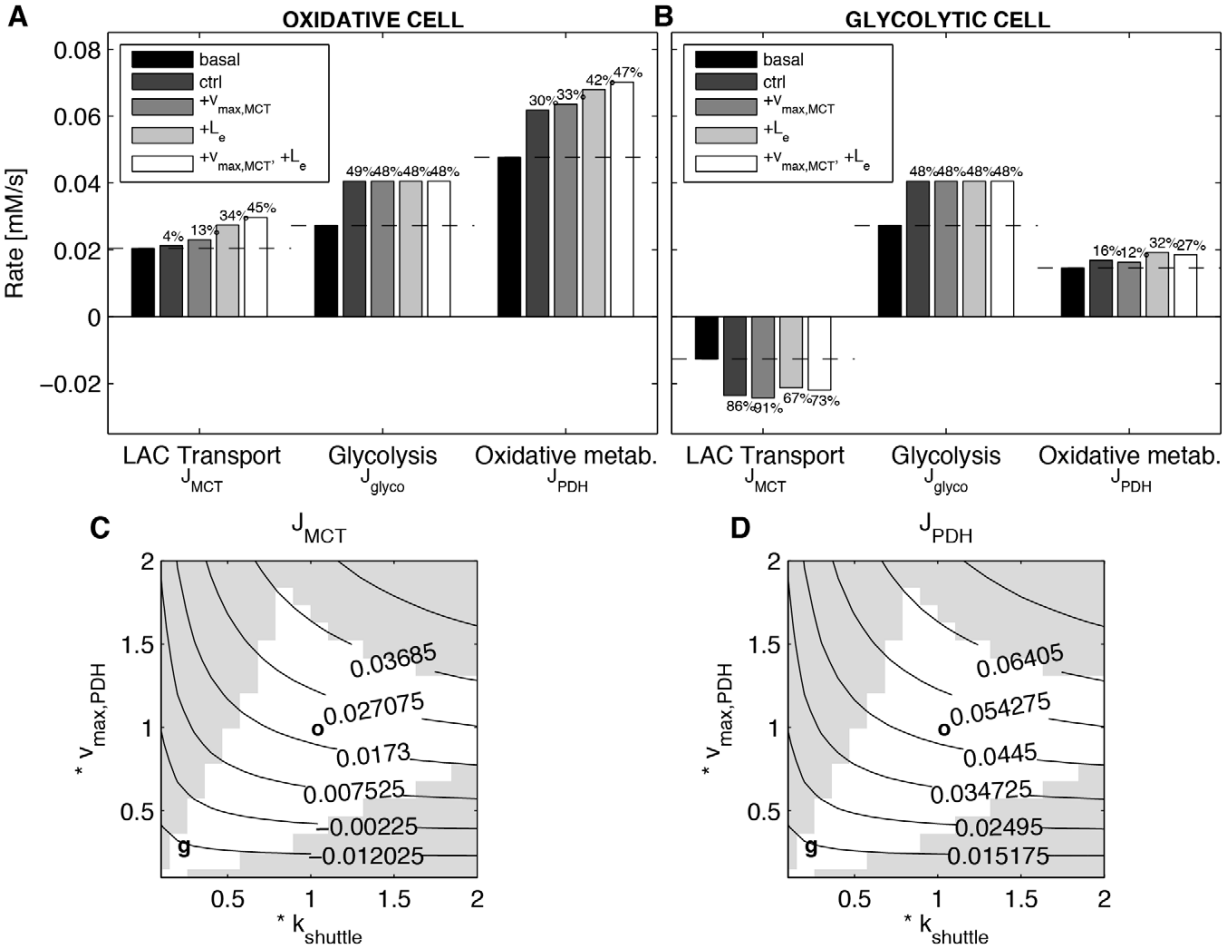
*J*_shuttle_ and *v*_max,PDH_ determine the occurrence of oxidative vs. glycolytic phenotype. (A) Oxidative phenotype: In the basal state (black), both lactate transport and glycolysis contribute to *J*_PDH_ (43% and 57% respectively [29]). We show the resulting steady state transport, glycolytic and *J*_PDH_ fluxes upon stimulation (very dark gray “*ctrl*-bar": +30% *v*_max,PDH_, +30% *k*_shuttle_, +48.5% *v*_max,glyco_; dark gray: +80% *v_max_*_,MCT_,, +30% *v*_max,PDH_, +30% *k*_shuttle_, +48.5% *v*_max,glyco_; light gray: +80% *L_e_*, +30% *v*_max,PDH_, +30% *k*_shuttle_, +48.5% *J*_max,glyco_; white: +80% *v*_max,MCT_, +80% *L_e_*, +30% *v*_max,PDH_, +30% *k*_shuttle_, +48.5% *v*_max,glyco_). (B) Glycolytic phenotype: In the basal state (black), parameters have been chosen such that lactate is taken out of the cell (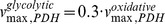, 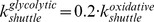). We show the resulting steady state transport, glycolytic and *J*_PDH_ fluxes upon stimulation (very dark gray “*ctrl*-bar": +30% *v*_max,PDH_, +30% *k*_shuttle_, +48.5% *v*_max,glyco_; dark gray: +80% *v*_max,MCT_, +15% *v*_max,PDH_, +0% *k*_shuttle_, +48.5% *v*_max,glyco_; light gray: +80% *L_e_*, +15% *v*_max,PDH_, +0% *k*_shuttle_, +48.5% *v*_max,glyco_; white: +80% *L_e_*, +80% *v*_max,MCT_, +15% *v*_max,PDH_, +0% *k*_shuttle_, +48.5% *v*_max,glyco_). See *Description of the model* for equations and *Choice of parameters* for parameters. (C–D) Basal lactate transport (C) and oxidative metabolism (D) when varying *v*_max,PDH_ and *k*_shuttle_. We considered 20 different values evenly spaced in the range 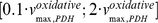 and 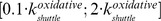, resulting in 400 simulations. For each simulation, we recorded the steady state value of *J*_MCT_ and *J*_PDH_. We show the resulting iso-curves (note that *v*_max,PDH_ and *k*_shuttle_ were normalized to their basal *oxidative* value, so that (1,1) (marked by ‘**o**’) corresponds to the parameters used in Fig. 2A and (0.2,0.3) (marked by ‘**g**’) corresponds to the parameters used in Fig. 2B). In non-shaded regions, the ratio 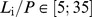 [32], 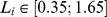 and 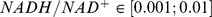 [34].

The model presented above was used to determine the changes in lactate flux across the plasma membrane as well as in the glycolytic and pyruvate dehydrogenase-catalyzed fluxes, when switching conditions from a basal to a stimulated state. For the basal state, the contribution of lactate-derived and glycolysis-derived pyruvate was set to 43% and 57%, respectively (or 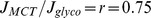), in accordance with the data of Nehlig and collaborators [29]. For the stimulated state, we allowed a 30% increase of the PDH maximal reaction rate 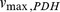 and of the mitochondrial NADH shuttling kinetic constant 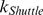, with a rather strong increase of 48.5% in the maximal glycolytic rate 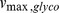 as well (see Text S1 for details). In addition, three scenarios were considered for lactate transport stimulation: 1) An increase in 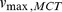, namely lactate maximal transport rate *via* MCTs, of 80% based on the data of Pierre et al. obtained in cultured neurons [35], due to MCT2 translocation to the cell membrane 2) An *in vivo* increase in the extracellular lactate concentration *L_e_* of 80% as reported by Hu and Wilson [36] 3) A concomitant increase in 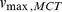 and *L_e_* of the same magnitude. Simulations allowed to determine the resulting fluxes for lactate transport, 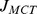, glycolysis, 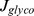, and oxidative metabolism, 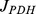. As can be observed in Fig. 2A, an enhancement of 30% in oxidative metabolism is obtained when increasing *v_max,glyco_*, *k_shuttle_* and *v_max,PDH_* alone (*ctrl*-bar). Further activating lactate transport capacity, 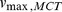, in a *stimulated* cell yields a slight increase in oxidative metabolism to +33%. Note that the increase in lactate flux remains relatively modest (+13%) in this condition. If instead an increase in the extracellular lactate concentration *L_e_* is taken into account, oxidative metabolism is further enhanced (+42%), with a significant increase in lactate flux (+34%). A combination of enhanced lactate transport capacity and extracellular lactate concentration leads to a 47% increase in oxidative metabolism with a concomitant 45% increase in lactate flux. It is noteworthy that in this last case, the contribution of each metabolic pathway approaches the basal state contributions (*r* = 0.73). Based on these metabolic responses, such a behavior appears characteristic of oxidative cells.

It is well known that a certain fraction of brain cells do not respond to a stimulation by importing lactate but rather by producing and releasing lactate. In order to reproduce this specific behavior, some of the parameters had to be modified. We found that reducing both the initial PDH reaction rate and the activity of the mitochondrial NADH shuttles leads to a strikingly different type of response. Fig. 2B presents the result of simulations performed when the basal values for these parameters were set to 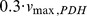 and 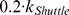 as compared to the simulations in Fig. 2A. Moreover, in the stimulated cell, the increase in PDH activity was limited to 15% and no increase in the kinetic constant of mitochondrial NADH shuttle was allowed (instead of +30% for both parameters in Fig. 2A, see Text S1 for details). In such case, a stimulation causing an activation of glycolysis and lactate transport produced a more limited increase in oxidative metabolism (+12%), but importantly yielded a prominent release of lactate (+91%), as indicated by the negative sign of the flux. Increasing the extracellular lactate concentration did not prevent lactate export (+67%), and oxidative metabolism was increased (+32%). In this case, the observed metabolic behavior rather corresponds to a typical predominantly glycolytic cell. Fig. 2C–D shows the basal lactate transport (C) and oxidative metabolism (D) obtained when varying both *v*_max,PDH_ and *k*_shuttle_, normalized to their basal oxidative reference values. On one hand, increases in *v*_max,PDH_ and/or *k*_shuttle_ can significantly increase pyruvate consumption by mitochondria, *J*_PDH_. This result can also be interpreted in the context of neural stimulation, showing that a stimulation of the NADH shuttles, especially the malate-aspartate shuttle, can usefully contribute to the increase in oxidative phosphorylation. On the other hand, decreasing *v*_max,PDH_ and/or *k*_shuttle_ leads to a glycolytic phenotype, with lactate secretion by the cell. Interestingly, while strongly reducing either *v*_max,PDH_ or *k*_shuttle_ is sufficient to switch from a basal oxidative to a glycolytic phenotype, these parameters need to be tuned together in order for the cell to preserve physiological lactate and pyruvate levels (non-shaded areas).

### Importance of lactate metabolism in sustaining oxidative metabolism

**Figure 3:**
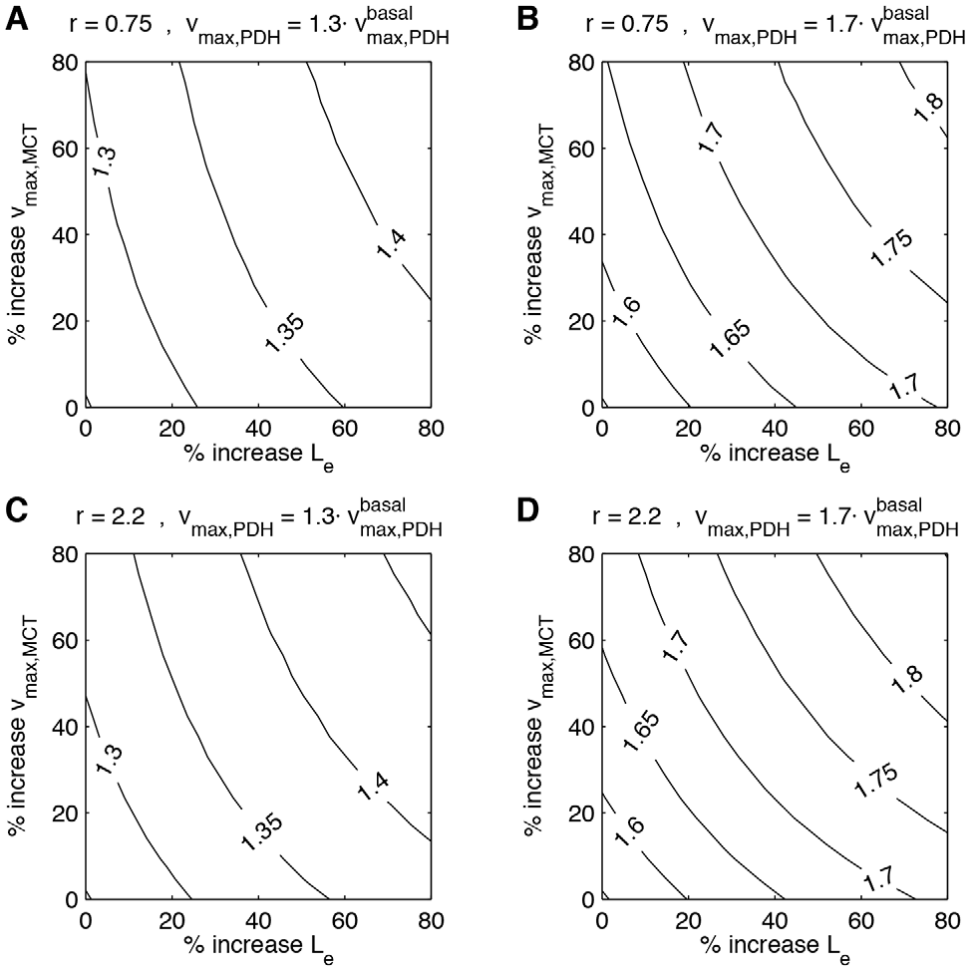
Importance of lactate transport for oxidative phosphorylation in oxidative cells. (A) As a starting point, it was assumed that lactate transport and glycolysis contribute 43% and 57%, respectively, to oxidative phosphorylation (*r* = 0.75 [29]). Lactate transport was then stimulated by increasing *v_max,MCT_* and *L_e_* in the range 0–80%. The iso-curves show *J*_PDH_ normalized to its basal value (as a measure of oxidative phosphorylation). Note that in parallel, *v_max,PDH_* and *k_shuttle_* were multiplied by a factor *f* = 1.3, while the glycolytic flux remained fixed to its basal level. In all regions, the ratio 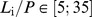 [32] and 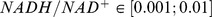 [34]. (B) As in (A), but *f* = 1.7. (C) As in (A), but *r* = 2.2 [28]. (D) As in (A), but *r* = 2.2 [28] and *f* = 1.7. See *Description of the model* for equations and *Choice of parameters* for parameters.

It could be observed in the previous simulations that lactate metabolism can significantly contribute to an enhancement in oxidative metabolism in cells with a predominantly oxidative phenotype (Fig. 2A). In these simulations, the presence of a strong glycolytic component was considered in parallel with lactate metabolism. However, evidence from the literature suggests that this is not the case in certain brain cell types, neurons in particular [14]. Thus, we investigated the capacity of lactate metabolism to sustain enhanced oxidative metabolism under *fixed* glycolytic conditions. Four scenarios were studied combining two lactate/glycolysis-supplied pyruvate ratios, *J_MCT_*/*J_glyco_* = *r*, at basal state, and two distinct levels of activation of the oxidative rate, 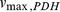: *r* = 0.75, +30% 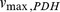 (Fig. 3A); *r* = 0.75, +70% 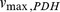 (Fig. 3B); *r* = 2.2, +30% 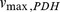 (Fig. 3C); and *r* = 2.2, +70% 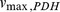 (Fig. 3D). In each case, a series of incremental oxidative fluxes were delineated, allowing to determine the combination of increased lactate transport capacity and extracellular lactate concentration that can sustain each oxidative flux (note that concentrations remained within physiological limits in all cases, cf. *Choice of parameters*). As can be seen, a rather wide range of increased oxidative rates can be accounted for by altering lactate transport capacity and extracellular lactate concentration. Importantly too, we see that increasing the PDH maximal capacity *v_max,PDH_* by 30% (respectively 70%) is not sufficient to yield an increase in oxidative metabolism *J*_PDH_ of 30% (respectively 70%). Hence, in order to be efficient under fixed glycolysis, the cell should not rely exclusively on the regulation of the PDH activity to increase its production of energy. In parallel, it should also enhance lactate transport, meaning that the latter could become limiting upon activation.

In order to better delineate under which range of basal conditions a change in lactate metabolism *alone* can satisfy physiological increases in oxidative rate, we varied the lactate/glycolysis-supplied pyruvate ratio, *J_MCT,0_*/*J_glyco,0,_* = *r*, between extreme cases (0.1 to 3.4 with increments of 0.3) together with increases γ in lactate flux of up to 80% (see Table 1; note that in this case, *v_max,glyco_*, *k_shuttle_* and *v_max,PDH_* were *not* enhanced, because the calculation is based here on Eq. (1)). As can be observed, increases in oxidative rate *J_PDH_* of up to 10% (white), between 10% and 29% (light gray), or even above 29% (dark gray) can be easily accounted by physiological increases in lactate flux for a wide range of basal conditions which encompasses most published values [15], [28], [29] (see also *Evaluation of model validity with in vitro and in vivo experimental data*).

### Evaluation of model validity with *in vitro* and *in vivo* experimental data

**Figure 4:**
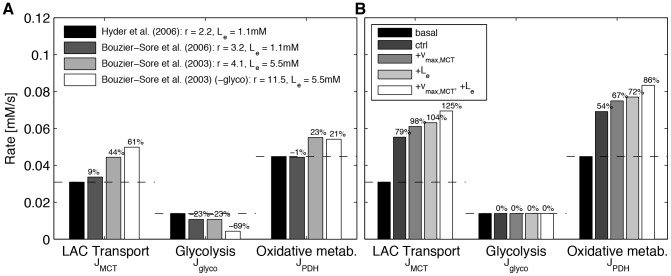
Model validity tested for different experimental conditions. (A) *Black*: *r* = *J_MCT_*/*J_glyco_* = 0.69/0.31 = 2.2 at basal state, as evaluated *in vivo* by Hyder et al. [28], with physiological values for intracellular lactate (L_i_) and pyruvate (P) concentrations (0.36 and 0.018 mM, respectively). L_e_ = 1.1 mM. *Dark gray*: *r* = *J_MCT_*/*J_glyco_* = 0.76/0.24 = 3.2, as observed *in vitro* by Bouzier-Sore et al. [15], with the experimentally used *L_e_* = 1.1 mM. *Light gray*: *L_e_* was increased to 5.5 mM, as described in the experiment by Bouzier-Sore et al. [16], resulting in a higher, but still plausible, value of 0.039 mM for intracellular pyruvate; *r* = 4.1. *White*: Same conditions as in *light gray*, but the glycolytic rate was lowered by 60%, resulting in a lower intracellular pyruvate concentration of 0.036 mM and a *J_MCT_*/*J_glyco_* ratio equal to 0.92/0.08 = 11.5, which matches experimental results by Bouzier-Sore et al. [16]. (B) Effect of lactate transport enhancement in the case of a basal *J_MCT_*/*J_glyco_* ratio (*r*) equal to 0.69/0.31 = 2.2, as evaluated *in vivo* by Hyder et al. [28], cf. black bar in (A). Basal state: *L_e_* = 1.1 mM (black). Stimulations: +30% *v*_max,PDH_, +30% *k*_shuttle_, +48.5% *v*_max,glyco_ (very dark gray, *ctrl*-bar); +0% *L_e_*, +80% *v*_max,MCT_, +70% *v*_max,PDH_, +70% *k*_shuttle_ (dark gray); +80% *L_e_*, +0% *v*_max,MCT_, +70% *v*_max,PDH_, +70% *k*_shuttle_ (light gray); +80% *L_e_*, +80% *v*_max,MCT_, +70% *v*_max,PDH_, +70% *k*_shuttle_ (white).

It is important to assess the validity of the model by testing whether it can account for published data. First, a ratio at basal state *r* = *J_MCT_*/*J_glyco_* = 0.69/0.31 = 2.2 as determined *in vivo* by Hyder et al. [28] was modeled with plausible flux rates for lactate transport, *J_MCT_*, glycolysis, *J_glyco_*, and oxidative metabolism, *J_PDH_* (Fig. 4A in black). Of interest, it gave rise to physiological intracellular lactate and pyruvate concentrations of 0.36 mM and 0.018 mM respectively, resulting in a lactate/pyruvate ratio of 19.5. Note that we used an extracellular lactate concentration of 1.1 mM and that the glycolysis flux was reduced by 49% to account for the data (compared to the oxidative cell of Fig. 2A). When considering *in vitro* data of Bouzier-Sore et al. [15] in cultured neurons, we used a ratio *r* = 0.76/0.24 = 3.2 with an extracellular lactate concentration of 1.1 mM as in the experimental condition (Fig. 4A, dark gray). Interestingly, increasing the extracellular lactate concentration to 5.5 mM, as used in Bouzier-Sore et al. [16], led to a higher but still quite plausible intracellular pyruvate concentration of 0.039 mM (Fig. 4A, light gray) and to a ratio *r* = 4.1, which is significantly lower than the corresponding experimental ratio (8). Reducing the glycolytic flux by 60% yielded a pyruvate concentration of 0.036 mM (Fig. 4A, white). In such case, we obtained a ratio *r* = 0.92/0.08 = 11.5, which matches the experimental results.

Finally, we decided to assess under conditions close to those observed *in vivo* whether lactate metabolism can account for a significant enhancement in oxidative rates following brain activation. Changes in lactate, glycolytic and oxidative fluxes following a stimulation were determined with the model using the experimental *in vivo* data of Hyder et al. [28], i.e. a ratio *r* = 0.69/0.31 = 2.2 and a resting extracellular lactate concentration of 1.1 mM (Fig. 4B). Three stimulated conditions were considered: increased lactate transport rate of 80% (Fig. 4B, dark gray), increased extracellular lactate concentration of 80% (Fig. 4B, light gray), and a combination of both (Fig. 4B, white). An increase in oxidative capacity of 70% was applied in parallel for all three stimulated conditions, as proposed by Hyder et al. [28]. As can be seen, the increase in lactate transport capacity or the extracellular lactate concentration led to an increase in oxidative flux of 67% and 72% respectively. But even more strikingly, a combination of the two parameters gave rise to an even more significant 86% increase in oxidative rate.

## Discussion

A critical question in neuroenergetics concerns the metabolic differences among the various brain cell types, and in particular between neurons and astrocytes. Several studies performed over the last 60 years using various approaches have documented distinctive features between these two cell types. Thus, using enzymatic analyses on individually isolated cells, Hydén and colleagues have suggested that neurons favor oxidative metabolism while glial cells rather exhibit a prominent glycolytic activity [37], [38]. With the advent of magnetic resonance spectroscopy applied *in vivo* or on purified preparations of both astrocytes and neurons in culture, several groups have confirmed the prominence of glycolysis in astrocytes and a more active tricarboxylic acid cycle in neurons [15], [39], [40], [41]. An important issue however is to determine which enzymatic elements could confer such a particular metabolic profile to these brain cell types. In this context, transcriptomic studies have all pointed out that astrocytes contain higher levels of several glycolytic enzymes than neurons [42], [43], [44]. In addition, studies on the distribution of isoforms of key proteins involved in lactate metabolism have been found to be consistent with astrocytes being glycolytic cells that export lactate, while neurons would be better equipped to oxidize it [25], [45], [46], [47]. Despite these pieces of evidence, it is still difficult to assess to what extent each specific element is critical to determine the particular metabolic profile observed in each brain cell type and how physiological metabolite concentrations are maintained. In this regard, our model provides a simple answer to this question. It revealed that altering the activity of only two components, the enzyme pyruvate dehydrogenase (PDH) and the mitochodrial NADH shuttling capacity, was sufficient to obtain two distinct metabolic phenotypes while preserving physiological concentrations. These phenotypes are characterized by their differential lactate metabolism. In the first case, cells use lactate as an oxidative energy substrate and are considered predominantly oxidative. The second phenotype exhibits lactate production and is associated with a rather glycolytic profile.

Interestingly, experimental evidence have been provided for a key role of these two enzymatic processes in the metabolic profile of brain cells. It was shown that neurons in culture oxidize readily substrates like lactate, which is not the case for cultured astrocytes that rather produce lactate [17]. But when astrocytes were treated with dichloroacetate, a substance that prevents PDH phosphorylation and enhances its activity, it promoted lactate oxidation and reduced its production from glucose. Indeed, it was shown that PDH activity is strongly inhibited constitutively in astrocytes *via* its phosphorylation while the neuronal PDH operates close to its maximal level [48] (ratio of 0.25 for *v*_max,PDH_ between astrocytes and neurons, similar to our value of 0.3 used in Fig. 2*B*). Such an observation indicates that the level of PDH activity is key to determine the oxidative capacity of the cell. In addition to the level of PDH activity, it was observed that astrocytes and neurons differ in terms of mitochondrial NADH shuttling activity, especially the malate/aspartate shuttle which is the most important NADH shuttle in the brain (ratio of 0.26 for *k*_Shuttle_ between astrocytes and neurons [49], similar to our value of 0.2 used in Fig. 2*B*). Thus, it was documented that astrocytes, in contrast to neurons, express very low levels of an essential component of the mitochondrial malate/aspartate NADH shuttle, the mitochondrial aspartate/glutamate transporter also known as aralar [49]. It was also demonstrated that glial cells have a low activity of the malate/aspartate shuttle [50], [51]. Moreover, when the mitochondrial aspartate/glutamate transporter was invalidated *via* a transgenic approach, cultured neurons from KO mice exhibited enhanced lactate production [52]. These data support the concept that a less efficient mitochondrial NADH shuttling mechanism, as observed in astrocytes, is associated with increased lactate production, as predicted by our model. Further analyses on the importance of PDH activity vs. mitochondrial NADH shuttling capacity also revealed that the level of active PDH determines the maximal oxidative capacity of the cell while varying mitochondrial shuttling rate allows rapid adjustments in oxidative rate (see Fig. S1 and Section *Effect of mitochondrial NADH shuttle vs. PDH parameters* in Text S2). In this sense, it has been shown that cytoplasmic Ca^2+^ levels control the activity of aralar [53], [54] and yield increased pyruvate levels, suggesting that adjustments of the NADH shuttle, *via* the control of cytoplasmic Ca^2+^ levels, act like a “gas pedal" [55]. Interestingly, it can be noticed that the oxidative and glycolytic profiles described in our model would fit well with the described behavior of a majority of neurons and astrocytes, respectively. However, it is not excluded that some neurons and/or astrocytes might exhibit a constitutively different metabolic profile or that they might alter their behavior as a function of activation, if their characteristics resemble those highlighted herein (see a modeling example of such a cell in Fig. S2 and in Section *Lactate export at rest – lactate consumption upon activation* in Text S2).

Recent evidence suggest that lactate represents an important oxidative energy substrate for the brain [56], [57] and more specifically for neurons *in vivo* [58]. These data add to numerous others obtained over decades in various *in vitro* preparations indicating that lactate represents a preferential oxidative energy substrate over glucose in neurons [15], [16], 17. A key question remaining was to what extent lactate oxidation can sustain the observed enhancement in oxidative metabolism occurring in brain cells following cerebral activation. In theory, lactate competes with glucose-derived pyruvate as an oxidative substrate, although we could demonstrate a substantial contribution of lactate to enhanced oxidative metabolism despite a strong rise in glycolysis in the stimulated condition. It was recently demonstrated however that cultured neurons cannot exhibit an enhancement in their glycolytic rate, as they lack an important component of the glycolytic regulatory cascade [14]. This situation is highly favorable to the use of lactate over glucose as a preferred oxidative substrate in these cells. Taking this point into account, our simulations demonstrate that under a wide range of initial glucose/lactate utilization ratios, most physiological increases in oxidative metabolism can be accounted for by realistic changes in lactate flux obtained by modifying either transport capacity, extracellular lactate concentration, or both. It is important to mention that following appropriate stimulation, both an increase in extracellular lactate concentration [36] and an increase in lactate transport capacity due to MCT2 membrane translocation in neurons [35] have been documented, providing physiological mechanisms for the modeling predictions. It was also important to consider other factors that can influence lactate flux under physiological conditions, in particular the pH changes known to occur following activation, and the LDH parameters. First of all, it was determined that upon stimulation, the observed pH changes would be favorable to both lactate transport and oxidation within an oxidative cell, as it would be the case in a neuron (see Fig. S3 and Section *Effect of intracellular and extracellular pH values* in Text S2). In contrast, it is interesting to notice that pH changes occurring with glutamate uptake in astrocytes prevent this glycolytic cell type to oxidize pyruvate and rather promote lactate release [59]. Concerning LDH, it could be shown that the responses obtained for an oxidative cell remain the same with different concentrations of total LDH (over a very wide range), suggesting that LDH activity does not represent a limiting factor for lactate oxidation at steady state (see Fig. S4 and Section *Effect of LDH parameters* in Text S2); however this does not preclude a physiological role of the distribution of LDH isoforms between neurons and astrocytes during fast transients. Similarly, using various equations for lactate transport via MCTs does not modify the results obtained, as long as the importance of the proton gradient is taken into account, a point that was neglected in previous modeling efforts ([60]; see Fig. S5 and Section *Effect of MCT parameters and alternative equation for MCT transport* in Text S2).

Finally, it was important to determine whether modeled cells could account for the experimental results obtained both *in vitro* and *in vivo* [15], [16], [28]. Indeed, it was the case but interestingly, modeling revealed that the glycolytic rate must be lower in cultured neurons compared to *in vivo* (cf. Fig. 4A). This is not surprising since the population of oxidative cells *in vivo* most likely displays a greater heterogeneity in terms of glycolytic rate than the selected population of neurons *in vitro*. Indeed, a low and rather uniform glycolytic rate was measured in cultured neurons as opposed to other cell types using an appropriate FRET nanosensor [61]. In contrast, the glycolytic rate measured for neurons in acute hippocampal slices by the same method was much more variable. Notwithstanding, the glycolytic rate in neurons (either in cultured neurons or in slices) was found to be on average much lower than in astrocytes [61], a feature which further emphasizes the importance of exogenous (astrocyte-derived) lactate vs. glucose-derived pyruvate to sustain oxidative metabolism in neurons. Another interesting prediction of our model concerns the importance of lactate transport capacity for lactate release by astrocytes and consumption by neurons. In fact, the capacity of astrocytes to provide lactate to surrounding cells upon stimulation seems directly dependent on both lactate transport and glycolysis capacities (cf. Fig. *2*B, dark gray bar, where glycolysis was increased by 48% and transport capacity by 80%, yielding a significant increase in lactate export of +91%). In parallel, neurons would rely on both increased lactate transport capacity and extracellular lactate concentration to support their energetic needs upon stimulation (cf. Fig. 3 and Table 1). In this regard, the recent observation that the expression of the monocarboxylate transporter MCT4 can be raised in astrocytes by nitric oxide, leading to enhanced lactate release, provides a mechanism to fulfil this predicted feature [62]. It remains to be determined whether a faster mechanism (e.g. membrane translocation) exists and would allow for rapid adaptation of lactate supply by astrocytes in register with neuronal activity, as is the case for MCT2 to facilitate lactate uptake in neurons [35].

Although the main findings of our modeling effort have been applied in the context of neuroenergetics, it is important to emphasize that implications extend far beyond. Indeed, cell-cell lactate shuttles have been reported in several other tissues, e.g. skeletal muscle [18], [22]. It is purported that the concept developed in the present work can be generalized to several physiological situations and may provide the main governing principles of cellular metabolic specialization as well as cell-cell metabolic cooperation in various biological organisms [63], [64], [65]. Furthermore, it could be applied to pathologies and explain certain characteristics of their development. For example, as observed by Warburg [2], many tumor cells exhibit aerobic glycolysis which gives them a key advantage to survive in a hypoxic environment, although others continue to rely on oxidative metabolism. In fact, our model provides a simple biochemical description of the Warburg effect. More recently, it was observed that oxidative tumor cells lying close to blood vessels consume lactate from more distant hypoxic tumor cells in order to spare glucose for these cells, thus favoring growth of the tumor [66]. In such case, our model offers a theoretical framework to describe the optimal conditions leading to disease progression. Based on such tools, perhaps it can be hoped that we could not only better understand factors that determine the evolution of various diseases, but it may also open up new therapeutic perspectives.

## Supporting Information

Figure S1PDH-metabolic flux as a function of the mitochondrial NADH shuttling rate *k*_shuttle_ and the maximal velocity of the PDH reaction, *v*_max,PDH_, which is proportional to the total amount of active PDH. The simulations in the gray areas did not match the following physiological constraints (cf. *Choice of parameters* in the main text): intracellular lactate concentrations 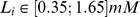 [30], [31], ratio 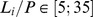 and redox state 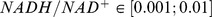 [34]. The black area represents the simulation with the parameters used for a typical oxidative cell (cf. Fig. 2A and Table S1).(TIF)

Figure S2NALS at rest, ANLS upon activation. (A) In the basal state (black), parameters are chosen such that lactate is taken out of the cell (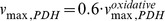, 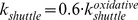, 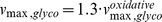). We show the resulting transport, glycolytic and *J*_PDH_ fluxes upon stimulation (dark gray: +80% *v*_max,MCT_, +100% *v*_max,PDH_, +70% *k*_shuttle_, +0% *v*_max,glyco_; light gray: +80% *L*_e_, +100% *v*_max,PDH_, +70% *k*_shuttle_, +0% *v*_max,glyco_; white: +80% *v*_max,MCT_, +80% *L*_e_, +100% *v*_max,PDH_, +70% *k*_shuttle_, +0% *v*_max,glyco_). (B) Same as (A), but glycolysis is also enhanced upon stimulation (+48.5% *v*_max,glyco_). Cf. Table S1 for the parameters of a typical oxidative cell.(TIF)

Figure S3Effect of pH on transport, glycolysis and PDH-metabolic flux. All the rates are normalized relative to their maximum. The intracellular proton concentration 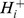 was varied within the range of [10^−3.6^; 10^−4.8^] mM; for the other parameters, refer to Table S1. The simulations in the gray areas did not match the following physiological constraints (cf. *Choice of parameters* in the main text): intracellular lactate concentrations 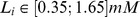 [30], [31], ratio 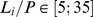 and redox state 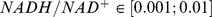 [34]. * represents the simulation with the parameters used for a typical oxidative cell (cf. Fig. 2A and Table S1).(TIF)

Figure S4Changing the LDH pool. (A) Effect of the total LDH pool on transport, glycolysis and PDH-metabolic flux. The simulations in the gray area did not match the following physiological constraints (cf. *Choice of parameters* in the main text): intracellular lactate concentrations 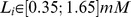 [30], [31], ratio 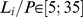 and redox state 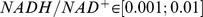 [34]. * represents the simulation with the parameters used for a typical oxidative cell ([LDH]_total_ = 3.2 mM, cf. Fig. 2A and Table S1). (B) [LDH]_total_ = 0.064 mM (compare with Fig. 2A of the main text where [LDH]_total_ = 3.2 mM). In the basal state (black), both glycolysis and lactate transport contribute to *J*_PDH_ (57% and 43% respectively). We show the resulting transport, glycolytic and *J*_PDH_ fluxes upon stimulation (dark gray: +80% *v*_max,MCT_, +30% *v*_max,PDH_, +30% *k*_shuttle_, +48.5% *v*_max,glyco_; light gray: +80% *L*_e_, +30% *v*_max,PDH_, +30% *k*_shuttle_, +48.5% *v*_max,glyco_; white: +80% *v*_max,MCT_, +80% *L*_e_, +30% *v*_max,PDH_, +30% *k*_shuttle_, +48.5% *v*_max,glyco_).(TIF)

Figure S5Effect of MCT parameters (transport equation in [60]). (A) Oxidative phenotype: In the basal state (black), both lactate transport and glycolysis contribute to *J*_PDH_ (43% and 57% respectively [29]). We show the resulting steady state transport, glycolytic and *J*_PDH_ fluxes upon stimulation (dark gray: +80% *v*_max,MCT_, +30% *v*_max,PDH_, +30% *k*_shuttle_, +48.5% *v*_max,glyco_; light gray: +80% *L*_e_, +30% *v*_max,PDH_, +30% *k*_shuttle_, +48.5% *v*_max,glyco_; white: +80% *v*_max,MCT_, +80% *L*_e_, +30% *v*_max,PDH_, +30% *k*_shuttle_, +48.5% *v*_max,glyco_). (B) Glycolytic phenotype: In the basal state (black), parameters are chosen such that lactate is taken out of the cell (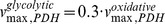, 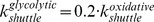). We show the resulting steady state transport, glycolytic and *J*_PDH_ fluxes upon stimulation (dark gray: +80% *v*_max,MCT_, +15% *v*_max,PDH_, +0% *k*_shuttle_, +48.5% *v*_max,glyco_; light gray: +80% *L*_e_, +15% *v*_max,PDH_, +0% *k*_shuttle_, +48.5% *v*_max,glyco_; white: +80% *L*_e_, +80% *v*_max,MCT_, +15% *v*_max,PDH_, +0% *k*_shuttle_, +48.5% *v*_max,glyco_). See Supp. Table S1 for the parameters of a typical oxidative cell. For lactate transport, we used 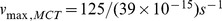 and the parameters in [60].(TIF)

Table S1 Parameters used in our simulations to compute the basal state of a typical oxidative cell (cf. Fig. 2A). For the references, refer to the bibliography in Text S1.
(DOC)

Text S1 Model description.
(DOC)

Text S2 Model robustness.
(DOC)
