# Supplementary figures and images for: Determinants of Brain Cell Metabolic Phenotypes and Energy Substrate Utilization Unraveled with a Modeling Approach
Source: PLoS Comput Biol. 2012 Sep 13;8(9):e1002686. doi: 10.1371/journal.pcbi.1002686 (PMC3441424; doi:10.1371/journal.pcbi.1002686)

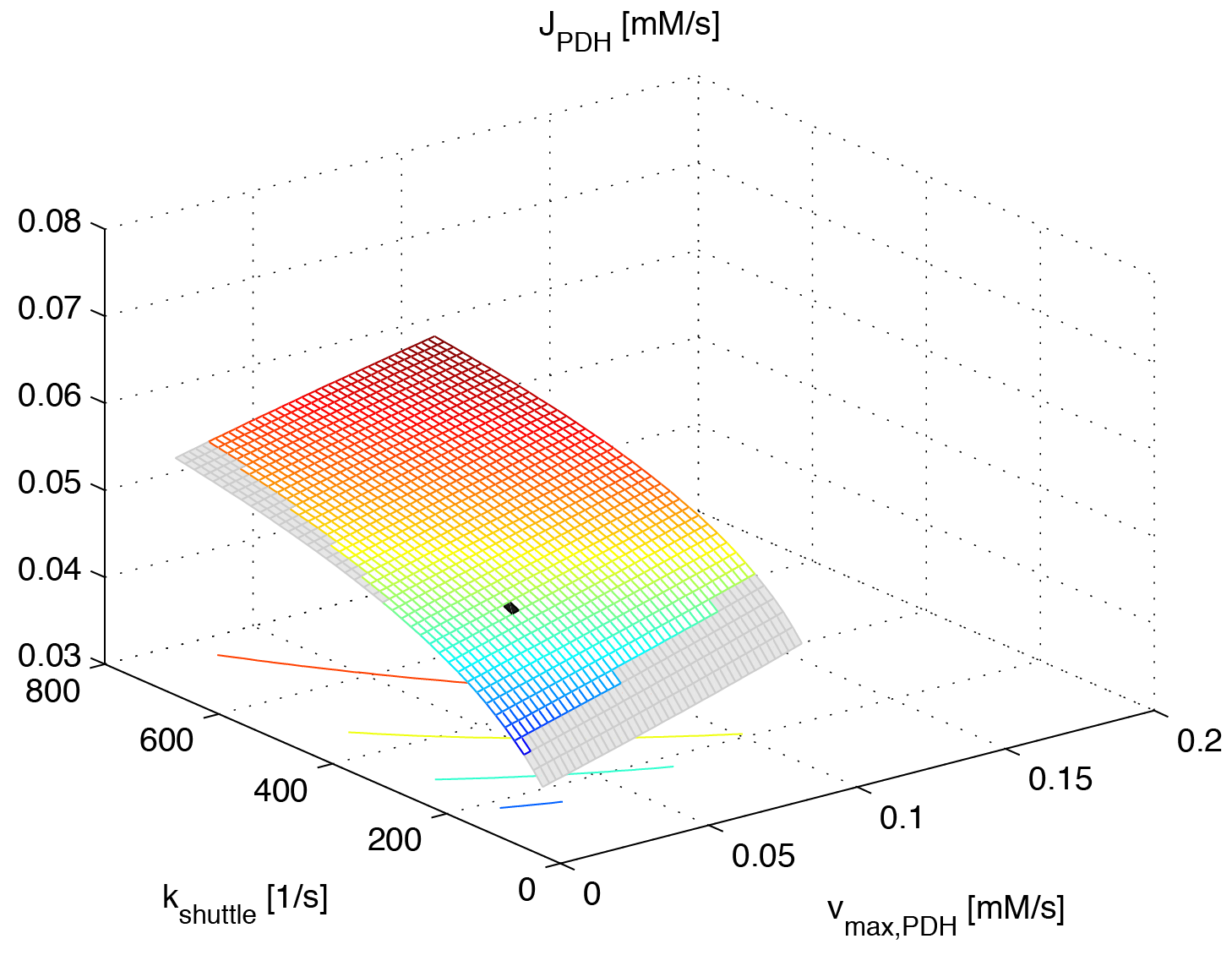

Supplement: Figure S1 — PDH-metabolic flux as a function of the mitochondrial NADH shuttling rate k shuttle and the maximal velocity of the PDH reaction, v max,PDH, which is proportional to the total amount of active PDH. The simulations in the gray areas did not match the following physiological constraints (cf. Choice of parameters in the main text): intracellular lactate concentrations [30], [31], ratio and redox state [34]. The black area represents the simulation with the parameters used for a typical oxidative cell (cf. Fig. 2A and Table S1). (TIF) [file pcbi.1002686.s001.tif]

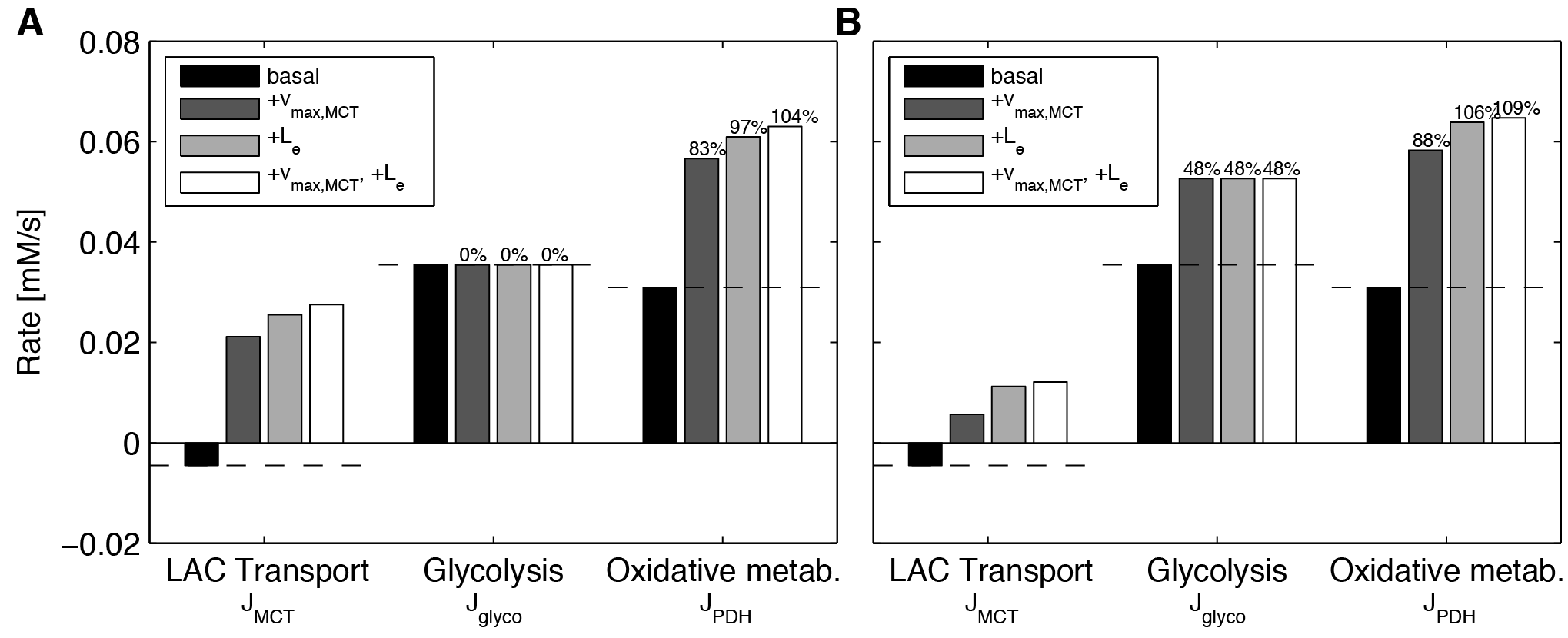

Supplement: Figure S2 — NALS at rest, ANLS upon activation. (A) In the basal state (black), parameters are chosen such that lactate is taken out of the cell (, , ). We show the resulting transport, glycolytic and J PDH fluxes upon stimulation (dark gray: +80% v max,MCT, +100% v max,PDH, +70% k shuttle, +0% v max,glyco; light gray: +80% L e, +100% v max,PDH, +70% k shuttle, +0% v max,glyco; white: +80% v max,MCT, +80% L e, +100% v max,PDH, +70% k shuttle, +0% v max,glyco). (B) Same as (A), but glycolysis is also enhanced upon stimulation (+48.5% v max,glyco). Cf. Table S1 for the parameters of a typical oxidative cell. (TIF) [file pcbi.1002686.s002.tif]

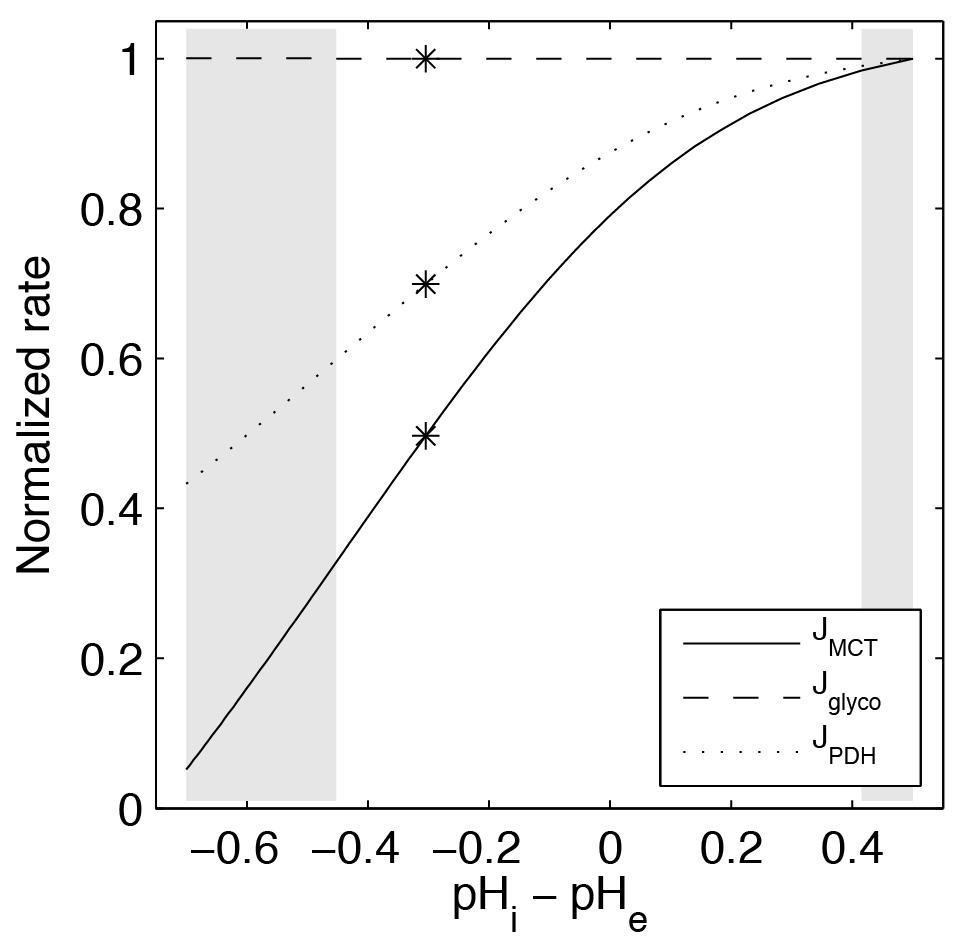

Supplement: Figure S3 — Effect of pH on transport, glycolysis and PDH-metabolic flux. All the rates are normalized relative to their maximum. The intracellular proton concentration was varied within the range of [10−3.6; 10−4.8] mM; for the other parameters, refer to Table S1. The simulations in the gray areas did not match the following physiological constraints (cf. Choice of parameters in the main text): intracellular lactate concentrations [30], [31], ratio and redox state [34]. * represents the simulation with the parameters used for a typical oxidative cell (cf. Fig. 2A and Table S1). (TIF) [file pcbi.1002686.s003.tif]

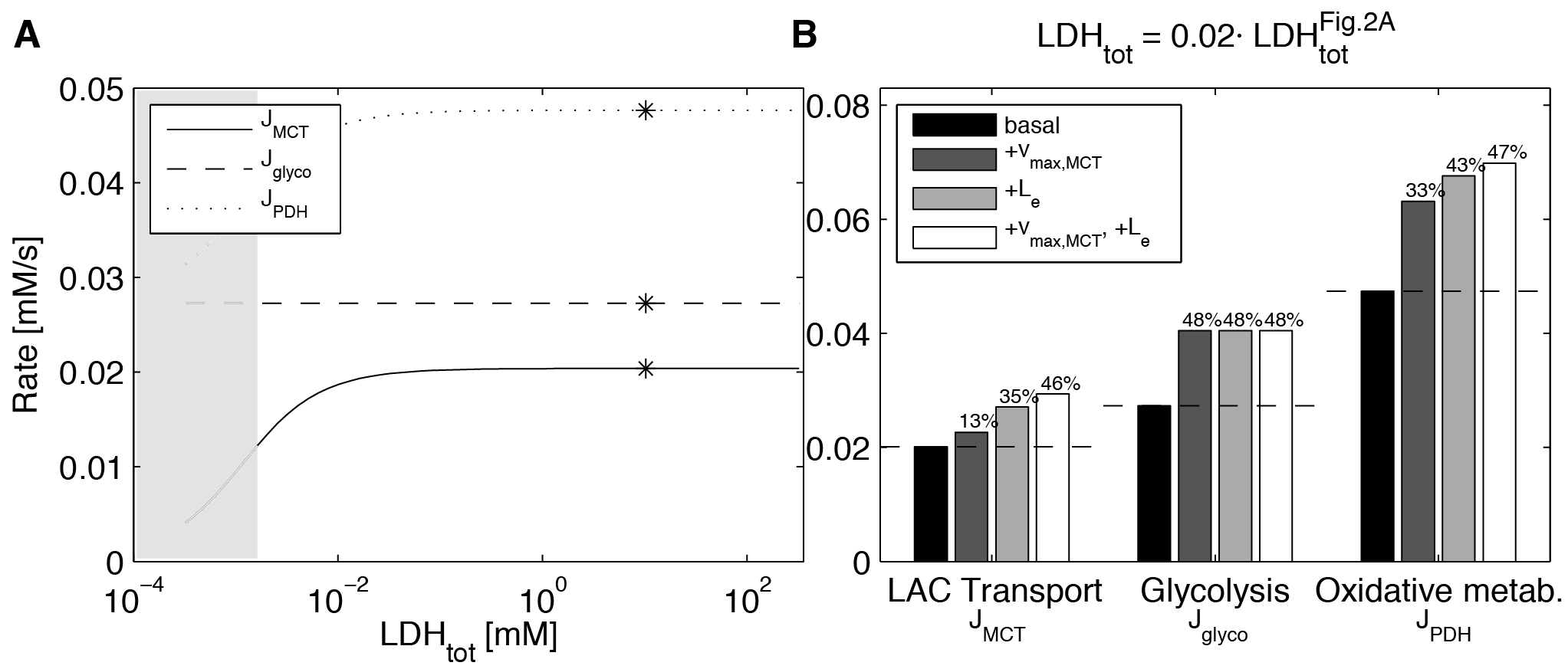

Supplement: Figure S4 — Changing the LDH pool. (A) Effect of the total LDH pool on transport, glycolysis and PDH-metabolic flux. The simulations in the gray area did not match the following physiological constraints (cf. Choice of parameters in the main text): intracellular lactate concentrations [30], [31], ratio and redox state [34]. * represents the simulation with the parameters used for a typical oxidative cell ([LDH]total = 3.2 mM, cf. Fig. 2A and Table S1). (B) [LDH]total = 0.064 mM (compare with Fig. 2A of the main text where [LDH]total = 3.2 mM). In the basal state (black), both glycolysis and lactate transport contribute to J PDH (57% and 43% respectively). We show the resulting transport, glycolytic and J PDH fluxes upon stimulation (dark gray: +80% v max,MCT, +30% v max,PDH, +30% k shuttle, +48.5% v max,glyco; light gray: +80% L e, +30% v max,PDH, +30% k shuttle, +48.5% v max,glyco; white: +80% v max,MCT, +80% L e, +30% v max,PDH, +30% k shuttle, +48.5% v max,glyco). (TIF) [file pcbi.1002686.s004.tif]

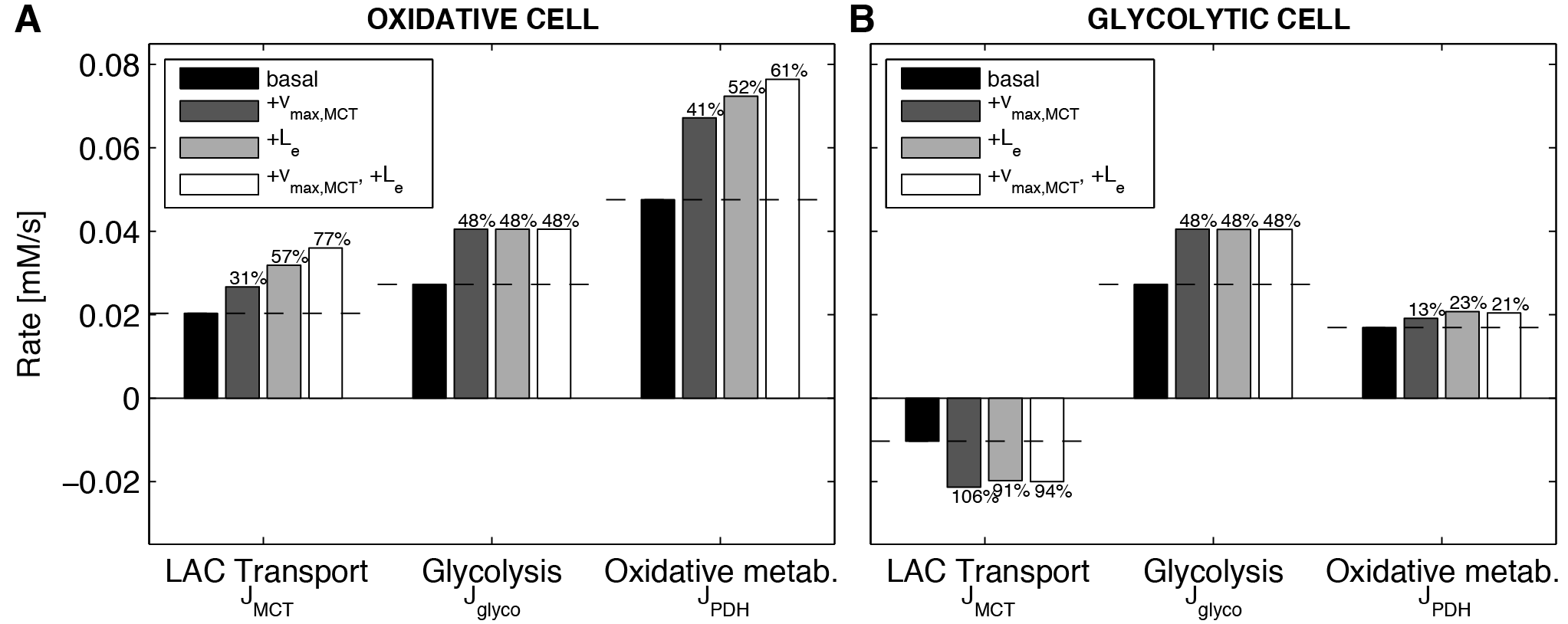

Supplement: Figure S5 — Effect of MCT parameters (transport equation in [60]). (A) Oxidative phenotype: In the basal state (black), both lactate transport and glycolysis contribute to J PDH (43% and 57% respectively [29]). We show the resulting steady state transport, glycolytic and J PDH fluxes upon stimulation (dark gray: +80% v max,MCT, +30% v max,PDH, +30% k shuttle, +48.5% v max,glyco; light gray: +80% L e, +30% v max,PDH, +30% k shuttle, +48.5% v max,glyco; white: +80% v max,MCT, +80% L e, +30% v max,PDH, +30% k shuttle, +48.5% v max,glyco). (B) Glycolytic phenotype: In the basal state (black), parameters are chosen such that lactate is taken out of the cell (, ). We show the resulting steady state transport, glycolytic and J PDH fluxes upon stimulation (dark gray: +80% v max,MCT, +15% v max,PDH, +0% k shuttle, +48.5% v max,glyco; light gray: +80% L e, +15% v max,PDH, +0% k shuttle, +48.5% v max,glyco; white: +80% L e, +80% v max,MCT, +15% v max,PDH, +0% k shuttle, +48.5% v max,glyco). See Supp. Table S1 for the parameters of a typical oxidative cell. For lactate transport, we used and the parameters in [60]. (TIF) [file pcbi.1002686.s005.tif]
