# Supplementary material for: Determinants of Brain Cell Metabolic Phenotypes and Energy Substrate Utilization Unraveled with a Modeling Approach
Source: PLoS Comput Biol. 2012 Sep 13;8(9):e1002686. doi: 10.1371/journal.pcbi.1002686 (PMC3441424; doi:10.1371/journal.pcbi.1002686)
Supplement: Table S1 — Parameters used in our simulations to compute the basal state of a typical oxidative cell (cf. Fig. 2A). For the references, refer to the bibliography in Text S1. (DOC) [file pcbi.1002686.s006.doc]

| **MCT transport** | **LDH metabolism** | **PDH metabolism** | **Glycolysis** | **Mitoch. shuttle** |
| --- | --- | --- | --- | --- |
| v_max,MCT_ = 0.29mM/s | v_max,LDH_^forward^ = 469mM/s | k_cat_ = 0.077/s | v_max,glyco_ = 0.041mM/s | k_shuttle_ = 300/s |
| L_e_ = 1.2mM | v_max,LDH_^reverse^ = 2035 mM/s | PDH_tot_ = 0.9mM | Glc = 1mM |  |
| H_i_ = 10^-4.0^mM | K_ia_ = 0.154mM | v_max,PDH_ = k_cat_ PDH_tot_ | K_Glc,glyco_ = 0.05mM |  |
| H_e_ = 10^-4.3^mM | K_iq_ = 0.001mM | K_P,PDH_ = 0.01mM | K_NAD+,glyco_ = 0.03mM |  |
| K_Hie_ = 0.7 H_e_ | K_mA_ = 0.000014mM |  |  |  |
|  | K_mQ_ = 0.0018mM |  |  |  |
|  | K_ib_ = 3892mM |  |  |  |
|  | K_ip_ = 0.0625mM |  |  |  |
|  | K_mB_ = 1.28mM |  |  |  |
|  | K_mP_ = 0.093mM |  |  |  |

For the references, refer to the bibliography in Text S1.
