# Supplementary material for: Determinants of Brain Cell Metabolic Phenotypes and Energy Substrate Utilization Unraveled with a Modeling Approach
Source: PLoS Comput Biol. 2012 Sep 13;8(9):e1002686. doi: 10.1371/journal.pcbi.1002686 (PMC3441424; doi:10.1371/journal.pcbi.1002686)
Supplement: Text S1 — Model description. (DOC) [file pcbi.1002686.s007.doc]

**Determinants of brain cell metabolic phenotypes and energy substrate utilization unraveled with a modeling approach**

**Aitana Neves^1*^, Robert Costalat^2*^ and Luc Pellerin^1^**

^1^Département de Physiologie, Université de Lausanne, Lausanne, Switzerland, ^2^UMMISCO, UPMC Université Paris 6 and IRD, Paris, France

*These authors contributed equally to the work

Address correspondence to [Luc.Pellerin@unil.ch](mailto:Luc.Pellerin@unil.ch)

**Supporting Information**

**Text S1. Model description**

We consider a model (cf. Fig. 1) where glucose (Glc) leads to pyruvate () *via* the glycolysis (), then pyruvate is metabolized by the PDH complex ($J_{PDH}$), leading to acetyl-CoA () which fuels the Krebs cycle in the mitochondria. Note that we define $J_{glyco}$ as the production of pyruvate by glycolysis, so value is twice the value of glucose consumption. On one hand, extracellular lactate () can be taken up by brain cells *via* MCTs (in which case we write$J_{MCT}>0$) and further metabolized by LDH into pyruvate ($J_{LDH}>0$); on the other hand, pyruvate can lead to lactate () and be extruded *via* the MCTs ($J_{MCT}<0$); we make no *a priori* assumption about the direction of the reaction catalyzed by LDH and transports *via* the MCTs. Furthermore, we take into account the NADH and NAD^+^ changes due to glycolysis, LDH-catalyzed reaction, and mitochondrial shuttles for transporting reducing equivalents from cytosolic NADH into the mitochondrial matrix (). Indeed, the role of these mitochondrial shuttles, malate-aspartate in particular, is to “transport” the NADH produced in the cytosol into the mitochondria. However, NAD^+^ and NADH cannot cross the mitochondrial membrane. To circumvent this, the shuttles rather transport electrons attached to malate. To do this, oxaloacetate in the cytosol is first reduced by NADH, yielding malate and NAD^+^ (this is what recycles NADH back to NAD^+^ in our model). Malate and the electrons it carries can then enter the mitochondria, while alpha-ketoglutarate is exported. Once inside, malate is transformed back to oxaloacetate by reducing mitochondrial NAD^+^ to NADH. Mitochondrial NADH is then free to transfer its electrons to the electron transport chain. Hence, the pools of mitochondrial and cytosolic NAD^+^/NADH are separated, but the presence of cytosolic NADH can yield the reduction of mitochondrial NAD^+^ to NADH.

Since we are interested in the cell behavior under various circumstances, we consider extracellular lactate as a parameter and we describe intracellular processes with a system of coupled differential equations:

where:

$J_{MCT}=v_{\max,MCT}\left(\frac{L_{e}\cdot H_{e}^{+}}{K_{Hie}+L_{e}\cdot H_{e}^{+}}-\frac{L_{i}\cdot H_{i}^{+}}{K_{Hie}+L_{i}\cdot H_{i}^{+}}\right)$, $J_{LDH}=\frac{v_{\max,LDH}^{forward}\cdot\left(NAD^{+}\cdot L_{i}-\frac{P\cdot NADH}{K_{eq}}\right)}{\left(\begin{matrix} K_{ia}K_{m_{B}}+K_{m_{B}}NAD^{+}+K_{m_{A}}L_{i}+NAD^{+}\cdot L_{i}+\frac{K_{ia}K_{m_{B}}}{K_{iq}}NADH+\frac{K_{m_{Q}}K_{ia}K_{m_{B}}}{K_{iq}K_{m_{P}}}P+\frac{K_{m_{B}}K_{m_{Q}}}{K_{m_{P}}K_{iq}}NAD^{+}\cdot P \\ +\frac{K_{ia}K_{m_{B}}}{K_{m_{P}}K_{iq}}P\cdot NADH+\frac{K_{m_{A}}}{K_{iq}}L_{i}\cdot NADH+\frac{NAD^{+}\cdot L_{i}\cdot P}{K_{ip}}+\frac{K_{m_{B}}K_{ia}}{K_{m_{P}}K_{iq}K_{ib}}L_{i}\cdot P\cdot NADH \end{matrix}\right)}$ $J_{glyco}=v_{\max,glyco}\left(\frac{Glc}{K_{Glc,glyco}+Glc}\right)\cdot\left(\frac{NAD^{+}}{K_{NAD^{+},glyco}+NAD^{+}}\right)$ ,

$$J_{shuttle}=k_{shuttle}\cdot NADH$$

$J_{PDH}=\frac{v_{\max,PDH}\cdot P}{K_{P,PDH}+P}$.

It can be noted that Eqs. (S1*b*) and (S1*c*) can be lumped together into one differential equation, with $NADH+NAD^{+}=$constant. Furthermore, the acetyl-CoA value can directly be obtained by a simple integration of $J_{PDH}$. Thus from a mathematical point of view, the dimension of the phase space is three.

It can also be noted that $v_{\max,glyco}$ and $Glc$are parameters of the model. For the sake of simplicity, the possible changes in $Glc$when a cell is stimulated are included in the $v_{\max,glyco}$ term.

The equation and parameters for lactate transport were taken from . In Text S2, we discuss another model for lactate transport .

The equation for the LDH reaction was taken from . It considers that $NAD^{+}$ has to bind LDH before lactate can do so, and that $NADH$ has to bind prior to pyruvate; this Ordered Bi-Bi mechanism is well documented for LDH . Note that the subscripts A, B, p, q refer to $NAD^{+}$, $L_{i}$, $P$, $NADH$, respectively. Also, according to Eq. (151) in ,

$$K_{eq}=\frac{v_{\max,LDH}^{forward}}{v_{\max,LDH}^{reverse}}\frac{K_{m_{P}}}{K_{m_{B}}}\frac{K_{iq}}{K_{ia}}$$

The constants *K_xx_* can be expressed in terms of the kinetic rates *k_x_* of the intermediary steps :

where we used the intermediary rates in . Note that LDH^*^, LDH^**^ are the NAD^+^-, NADH-activated LDH, respectively. Also, according to , $K_{ia}=\frac{k_{-1}}{k_{1}}=0.07 \text{mM}$ and $K_{iq}=\frac{k_{4}}{k_{-4}}=0.001 \text{mM}$ for heart LDH namely, LDH_1_ (in our simulations, we took the maximal rates in the forward and reverse direction from and adjusted *K_ia_* to $K_{ia}=0.15\text{mM}$ in order to get closer to $K_{eq}\approx1.1\cdot10^{-4}$, which is the generally acknowledged value at pH = 7.0; see Text S2). Finally, Eq. (150) in gives two different formula for the total enzyme concentration as a function of the *k_x_*. Hence, the remaining free parameters, *k_-1_* and *k_4_*, are chosen such that these two formula yield the same total enzyme concentration. The values of the two kinetic constants of the reaction which occurs between complexes, namely $\left[LDH^{*}-L_{i}\right]\begin{matrix} \xrightarrow{} \\ \leftarrow\end{matrix}\left[LDH^{**}-P\right]$ were somewhat difficult to choose, since this reaction may include several intermediary steps ; however, it is acknowledged that the equilibrium constant for these complexes is near unity , and altering the kinetic constants while keeping this equilibrium constant close to unity resulted in very slight changes in the results.

The PDH-catalyzed reaction was considered to be irreversible and to follow Michaelis-Menten-type kinetics (parameters from ).

Glycolysis was also modeled using the irreversible Michaelis-Menten formalism, considering both glucose and NAD^+^ as substrates .

Only stationary states were considered. See Table S1 for the parameters of a typical oxidative cell in its basal state (see also Fig. 2A).

Although there are no experimental data about increases of these parameters upon stimulation, numerous data about *in vivo* changes of oxygen consumption (CMRO_2_) and glucose consumption (CMR_Glc_) lead to likely values, which can then be varied to test the robustness of the results.

First, physiological increases of CMR_Glc_ range in the [+17%;+51%] interval (for reviews, see ), while CMR_Glc_ increase can be as high as [+100%;+400%] during seizures . Thus it is probable that only part of the cells undergo enhanced glycolysis under physiological stimulation. Therefore an increase in the maximum glycolytic rate of individual cells of about 50% seems quite plausible.

Second, increases of PDH and NADH shuttle rates are strongly linked to Krebs cycle and oxidative phosphorylation rates, thus to CMRO_2_ (see e.g. ). Reported CMRO_2_ increases upon physiological stimulations are various, ranging in the [+5%;+47%] interval , bearing in mind that CMRO_2_ measures oxygen consumption in a tissue voxel including both oxidative and glycolytic cells. During seizures, CMRO_2_ can increase up to +267% . Although CMRO_2_ increases need not fully match increases in PDH maximal rate and NADH shuttle kinetic constant, a +30% increase of these parameters is a plausible value. In addition, all the above-mentioned studies indicate that physiological CMRO_2_ increases are smaller than, or sometimes equal to, CMR_Glc_ increases, which is consistent with the chosen parameter values.

References

1. Aubert A, Costalat R, Magistretti P, Pellerin L (2005) Brain lactate kinetics: Modeling evidence for neuronal lactate uptake upon activation. Proc Natl Acad Sci U S A 102: 16448-16453.

2. Simpson IA, Carruthers A, Vannucci SJ (2007) Supply and demand in cerebral energy metabolism: the role of nutrient transporters. J Cereb Blood Flow Metab 27: 1766-1791.

3. Kuby SA (1990) A Study of Enzymes, Volume I: CRC Press, Boca Raton. 472 p.

4. Gutfreund H (1995) Kinetics for the Life Sciences: Receptors, Transmitters and Catalysts: Cambridge University Press, Cambridge. 346 p.

5. Zhadin N, Gulotta M, Callender R (2008) Probing the role of dynamics in hydride transfer catalyzed by lactate dehydrogenase. Biophys J 95: 1974-1984.

6. Segel IH (1975) Enzyme kinetics: Behavior and analysis of rapid equilibrium and steady state enzyme systems: Wiley-Interscience, New York. 957 p.

7. O'Brien J, Kla K, Hopkins I, Malecki E, McKenna M (2007) Kinetic parameters and lactate dehydrogenase isozyme activities support possible lactate utilization by neurons. Neurochem Res 32: 597-607.

8. Seifert F, Golbik R, Brauer J, Lilie H, Schröder-Tittmann K, et al. (2006) Direct kinetic evidence for half-of-the-sites reactivity in the E1 component of the human pyruvate dehydrogenase multienzyme complex through alternating sites cofactor activation. Biochemistry 45: 12775-12785.

9. Berg J, Tymoczko J, Stryer L (2010) Biochemistry. New York: W. H. Freeman. 1120 p.

10. Bouzier-Sore A-K, Voisin P, Bouchaud V, Bezancon E, Franconi J-M, et al. (2006) Competition between glucose and lactate as oxidative energy substrates in both neurons and astrocytes: a comparative NMR study. Eur J Neurosci 24: 1687-1694.

11. Shulman RG, Rothman DL (1998) Interpreting functional imaging studies in terms of neurotransmitter cycling. Proceedings of the National Academy of Sciences of the United States of America 95: 11993-11998.

12. Gjedde A (2001) Brain energy metabolism and the physiological basis of the hemodynamic response. In: Jezzard P, Matthews P, Smith S, editors. Functional Magnetic Resonance Imaging: Methods for Neuroscience. Oxford: Oxford University Press. pp. 37-65.

13. Dienel G (2002) Energy generation in the central nervous system. In: Edvinsson L, Krause D, editors. Cerebral blood flow and metabolism. Philadelphia: Lippincott Williams and Wilkins. pp. 140-161.

14. Fox PT, Raichle ME, Mintun MA, Dence C (1988) Nonoxidative glucose consumption during focal physiologic neural activity. Science 241: 462-464.

15. Gjedde A, Marrett S (2001) Glycolysis in neurons, not astrocytes, delays oxidative metabolism of human visual cortex during sustained checkerboard stimulation in vivo. J Cereb Blood Flow Metab 21: 1384-1392.
