# Supplementary material for: Determinants of Brain Cell Metabolic Phenotypes and Energy Substrate Utilization Unraveled with a Modeling Approach
Source: PLoS Comput Biol. 2012 Sep 13;8(9):e1002686. doi: 10.1371/journal.pcbi.1002686 (PMC3441424; doi:10.1371/journal.pcbi.1002686)
Supplement: Text S2 — Model robustness. (DOC) [file pcbi.1002686.s008.doc]

**Determinants of brain cell metabolic phenotypes and energy substrate utilization unraveled with a modeling approach**

**Aitana Neves^1*^, Robert Costalat^2*^ and Luc Pellerin^1^**

^1^Département de Physiologie, Université de Lausanne, Lausanne, Switzerland, ^2^UMMISCO, UPMC Université Paris 6 and IRD, Paris, France

*These authors contributed equally to the work

Address correspondence to [Luc.Pellerin@unil.ch](mailto:Luc.Pellerin@unil.ch)

**Supporting Information**

**Text S2. Model robustness**

**Effect of mitochondrial NADH shuttle vs. PDH parameters**

The mitochondrial malate-aspartate shuttle has been shown to be mainly active in neurons and a recent study shows that the total amount of active PDH is also higher in these cells as compared to astrocytes. We thus wanted to investigate how these two processes contribute to oxidative phosphorylation and may modulate it.

As shown in Fig. S1, an increase in the mitochondrial shuttling rate yields an increase in oxidative phosphorylation (which we assume proportional to *J*_PDH_). Interestingly, small changes in the mitochondrial shuttling rate induce a greater oxidative response than small changes in the total amount of active PDH (proportional to *v*_max,PDH_). However, the effect due to an increase in mitochondrial shuttle also saturates faster. It therefore appears that modulating the mitochondrial shuttling rate is an efficient means to increase oxidative phosphorylation upon high demand, while modulating the total amount of active PDH determines the maximal oxidative capacity of the cell (i.e. the saturation level of *J*_PDH_ when changing the mitochondrial shuttling rate). This suggests that oxidative cells should have higher levels of active PDH than their glycolytic counterparts, as was indeed recently observed .

**Lactate export at rest – lactate consumption upon activation**

In this section, we report a scenario where a cell can be a lactate producer at rest, and consume lactate upon activation (Fig. S2A), even if glycolysis is enhanced (Fig. S2B). In the basal state, *v_max,glyco_* was enhanced (+30%), while *v_max,PDH_* and *k_shuttle_* were reduced compared to the oxidative cell model presented in Fig. 2A (-40% in both cases)*.* Hence, such a scenario would be consistent with a cell exhibiting NALS at rest and ANLS upon stimulation. It remains to be determined however if such cells do exist, how abundant they are and in which type of cognitive processes (e.g. sensori-motor) they are engaged.

**Effect of intracellular and extracellular pH values**

It is well known that cellular membranes of biological systems exhibit a gradient of proton concentration and that it is important for neuronal excitability . In fact, one should consider the transmembrane difference of H^+^ ions electrochemical potential; this electrochemical potential is defined as

$$\tilde{\mu}_{H^{+}}=\mu_{H^{+},0}+\text{R}T \text{ln[}H^{+}\text{]}+z_{H^{+}}\text{F}V=\mu_{H^{+},0}+\text{R}T \text{ln[}H^{+}\text{]}+\text{F}V$$

where $\mu_{H^{+},0}$ is the standard chemical potential of H^+^ ions, R the Gas constant, *T* the absolute temperature, *V* the electric potential, and F the Faraday constant. A straightforward calculation shows that H^+^ ions tend to enter the cell if an only if $61\times(pH_{i}-pH_{e})>V_{m}$, where $V_{m}$ is the membrane potential expressed in mV, $pH_{i}$ and $pH_{e}$ are intracellular and extracellular pH respectively. At steady state, this passive transport is balanced by active transport mechanisms.

In the brain, several studies have investigated the pH at rest and under stimulated conditions. Extracellular pH was found to be on average around 7.3 , while intracellular pH was generally more acid, close to 7.0. Indeed, *in vitro* studies reported an astrocytic and neuronal pH of 6.98-7.24 and 6.93-7.07 , respectively. Upon stimulation, studies in cultured hippocampal neurons have revealed that depolarization induces *intracellularly* a transient acid response followed by a large alkaline shift . Interestingly, at the *extracellular* level, the pH response in hippocampal CA1 area upon neuronal activity consisted in an initial alkalinization, followed by a slower acidification , both *in vivo* and *in vitro*. The observed transient alkalinization could be related to the dip in extracellular lactate observed by Hu and Wilson . Of interest too, Xiong and Stringer showed that blocking glial depolarization with barium increased the initial alkalinization “dip” in both CA1 and dentate gyrus, suggesting that glial cells contribute to the acidification of the extracellular medium, e.g. by the release of lactate. Moreover, depolarization-induced alkalinization of neurons , and acidification of the extracellular medium could further favour the import of lactate by neurons during e.g. prolonged neuronal activity. Lactate could therefore sustain neuronal energetic needs, as long as the lactate-transport-induced neuronal acidification does not counter-balance the depolarization-induced neuronal alkalinization (note that neuronal acidification could be slowed down by acid/base transporters or by the cytosolic protons being massively consumed by the mitochondria to synthesize ATP). On the astrocytic side, it has also been shown that glutamate transport diminishes cytosolic and mitochondrial pH, hence reducing ATP production due to the quasi-absence of a proton gradient between the mitochondria and cytosol, and favouring the release of the produced lactate, since the cytosolic pH dropped to 7.2 , below extracellular pH .

In the following, we investigated how the changes in pH affect the transport and metabolic fluxes within our model. From a thermodynamical perspective, it appears that pH changes need to be considered when investigating the directionality of lactate transport. Indeed, the latter will depend on the electrochemical gradient of lactate (L) and H^+^ ions. If we consider the electrochemical potential $\tilde{\mu}$, we have:

where and $\mu_{H^{+},0}$ are the standard chemical potentials, *V* the electric potential, as above. Since the lactate ion has only one elementary negative charge, , and since $z_{H+}\text{=+1}$, it follows that

.

Hence, the direction of the net transport of lactate by the MCTs is determined by the product [L].[H^+^], where both terms are important.

In order to test the effect of pH within our modeling framework, we performed simulations on

an oxidative cell for which the extracellular pH was kept constant while the intracellular pH was varied (as a mean to vary the proton gradient). However, modeling precisely the effects of cytosolic pH ($pH_{i}$) on LDH kinetics has not yet been achieved to our knowledge ; in fact, it is a very complex problem since pH variations can alter enzyme kinetics at every intermediary step. We chose to take into account the effect of $pH_{i}$ by modifying the apparent equilibrium constant, $K_{eq}$, of the reaction, replacing by ($H_{i}^{+}$ being expressed in mM). This change takes into account the thermodynamic constraints, since the equilibrium constant of the fully-described LDH-catalyzed reaction can then be expressed as , namely the value obtained at 38°C by Williamson, Lund and Krebs , from which Ido et al. derived the apparent constant $K_{eq}$ at $pH_{i}=7.0$ . Furthermore, we verified that altering individual kinetic rates, whenever the thermodynamic constraint is fulfilled, only slightly modifies the results. This latter observation is consistent with the experimental data reported by Vinnakota et al. , which show that the apparent maximum velocity (in the reverse direction) of this reaction would be proportional to ; thus, decreasing $pH_{i}$ to 6.7 or increasing $pH_{i}$ to 7.4 would result in modifications of the maximum velocity of about 5%. In summary, we can confidently assume that the main effect of altering $pH_{i}$ on LDH kinetics is the modification of the apparent equilibrium constant, at least in the studied domain.

As can be seen in Fig. S3, glycolysis (*J_glyco_*) was pH-independent as expected. Lactate transport (*J_MCT_*) was however pH-dependent, and increased with the proton gradient. Interestingly, lactate import occurred even when the proton gradient was highly unfavorable (pH_i_-pH_e_<-0.3). It is also important to note that if upon stimulation the difference pH_i_-pH_e_ increases, as suggested by , it should increase both transport *and* oxidative phosphorylation (*J_PDH_*) according to our model. Close to saturation (pH_i_-pH_e_>0.42), the levels of lactate increased above 1.65mM and hence, the results were no longer considered to be physiologically relevant. For pHi-pHe<-0.45, lactate levels were below 0.35mM, hence not satisfying our physiological constraints either (note that intracellular lactate concentrations in this region were within the range of [0.23;0.35[mM). Between these points (non-shaded area), lactate levels lied between 0.35-1.52mM, pyruvate levels between 0.015-0.24mM, L_i_/P between 6.5-24.4 and NADH/NAD^+^ between 0.0019-0.0031.

**Effect of LDH parameters**

We wanted to investigate the robustness of the model to changes in LDH-related parameters, since these were kept constant for our simulations reported in the main text. In that case, we considered an LDH concentration of the order of the mM, corresponding to about 35g/L (LDH molecular weight was taken as ~35000Da). This high concentration of LDH is most probably not uniform, but found locally in the cell, in regions where the protein might be confined. This is in agreement with the idea of “enzyme organization”: cells are not homogeneous entities containing metabolites and enzymes but rather, enzymes are (i) confined to organelles, (ii) bound to membranes, (iii) bound to the cytoskeleton or (iv) associated to other proteins for metabolic channeling (*Metabolic Channeling in Organized Enzyme Systems: Experiments and Models* in ). Therefore, though measured *in vitro* enzyme concentrations are often of the order of the μM for metabolite concentrations of the order of the mM, *in vivo* enzyme concentrations might actually be of the order of the mM locally, just like metabolite concentrations.

However, it is not yet clear if “enzyme organization” indeed applies to the LDH or if it is distributed (more or less) uniformly in the cell. In muscle, some data suggest that the LDH might be localized in “subcellular particulate fractions” . Although it has been suggested that LDH could be situated in the matrix of the muscle mitochondria, there is no consensus about this point (for a review, see ). Several authors suggested that muscle LDH is localized in the mitochondria intermembrane space, and even can be attached to the inner membrane . However, no clear evidence has yet been found in the brain to our knowledge. Since *in vitro* studies report the maximal rate normalized per mg protein and that, to our knowledge, *in vivo* measurements of the LDH pool have not been reported, we considered in our simulations several pools of total LDH, ranging from 0.32μM to 320mM. In Fig. S4A, we see that despite changing the total amount of LDH by a factor ~1.6x10^5^ (within the physiological non-shaded curves), the mitochondrial flux *J_PDH_* and lactate transport only vary by a factor 1.2 and 1.5, respectively, showing great robustness. The glycolytic rate is unaffected by the amount of the LDH pool.

Importantly too, as we see in Fig. S4B, the model responds very similarly upon activation at low concentrations of LDH ([LDH]_total_ = 0.064mM, compared with Fig. 2A of the main text where [LDH]_total_ = 3.2mM). Thus, although the value of the *in vivo* LDH concentration remains debated, our study shows that at steady state, the cell responds in a very similar fashion upon activation, almost independently of the total LDH pool. The LDH concentration, as well as the distribution of LDH isoforms, could however play an important role during transient responses, when the system is not at steady state.

**Effect of MCT parameters and alternative equation for MCT transport**

In our simulations, lactate transport by the MCTs was modeled according to :

$J_{MCT}=-v_{\max,MCT}\left(\frac{L_{i}\cdot H_{i}^{+}}{K_{Hie}+L_{i}\cdot H_{i}^{+}}-\frac{L_{e}\cdot H_{e}^{+}}{K_{Hie}+L_{e}\cdot H_{e}^{+}}\right)$.

The initial purpose of our study was to investigate which metabolic parameters are key to determine the direction of the lactate flux. It did not aim at predicting exact stationary metabolite concentrations or reproduce measured cerebral metabolic rates. Rather, given a cell with both oxidative and glycolytic capacity, we investigated which conditions drive it rather towards one metabolic behavior or the other. Thus, for simplicity, we used the MCT description provided by .

Still, considering that later studies have proposed a more complex transport model , we also tested whether our results still hold when using the more complex lactate transport equation:

$$J_{MCT}^{Simpson}=-v_{\max,MCT}\frac{L_{e}\cdot H^{+}\left({}^{n}K_{L}+L_{i}\cdot H^{+}\right)-L_{i}\cdot H^{+}\left({}^{n}K_{L}+L_{e}\cdot H^{+}\right)}{{}^{n}K_{L}^{2}\cdot{}_{L}^{n}R_{oo}+{}^{n}K_{L}\cdot{}_{L}^{n}R_{oi}\cdot L_{e}\cdot H^{+}+{}^{n}K_{L}\cdot{}_{L}^{n}R_{io}\cdot L_{i}\cdot H^{+}+{}_{L}^{n}R_{ee}\cdot L_{e}\cdot H^{+}\cdot L_{i}\cdot H^{+}}$$

As can be seen in Fig. S5, the oxidative and glycolytic cell types were robust to the transport equation that was used (in these simulations, we used the values reported in for the transport-related parameters, and we adjusted *v_max,MCT_* to ensure that the ratio $r=J_{MCT}/J_{glyco}=0.75$ in the oxidative cell). Note that this equation yields a similar trend of net lactate transport as a function of intracellular lactate compared to the equation we used . However, it is important to point out that the study by Simpson et al. does not consider the proton gradient, since pH is considered to be the same intra- and extracellularly. Importantly, in section *Effect of intracellular and extracellular pH values* of this supplement, we have recalled that the direction of the net transport of lactate by the MCTs is actually determined by the product [L].[H^+^] and hence, the proton gradient should not be neglected. Such an observation certainly explains at least in part the divergent conclusion reached by Simpson et al. concerning the direction of lactate flux in each cell type. In summary, *(i)* the choice of the kinetic equation for MCTs, namely from or , does not alter our main conclusions about the direction of lactate transport; *(ii)* however, taking into account the pH transmembrane gradient, which is achieved in but not in , is highly recommendable for modeling lactate transport, due to thermodynamic constraints.

References

1. Berkich DA, Ola MS, Cole J, Sweatt AJ, Hutson SM, et al. (2007) Mitochondrial transport proteins of the brain. J Neurosci Res 85: 3367-3377.

2. McKenna MC, Waagepetersen HS, Schousboe A, Sonnewald U (2006) Neuronal and astrocytic shuttle mechanisms for cytosolic-mitochondrial transfer of reducing equivalents: current evidence and pharmacological tools. Biochem Pharmacol 71: 399-407.

3. Ramos M, del Arco A, Pardo B, Martínez-Serrano A, Martínez-Morales JR, et al. (2003) Developmental changes in the Ca2+-regulated mitochondrial aspartate-glutamate carrier aralar1 in brain and prominent expression in the spinal cord. Brain Res Dev Brain Res 143: 33-46.

4. Xu Y, Ola MS, Berkich DA, Gardner TW, Barber AJ, et al. (2007) Energy sources for glutamate neurotransmission in the retina: absence of the aspartate/glutamate carrier produces reliance on glycolysis in glia. J Neurochem 101: 120-131.

5. Halim ND, Mcfate T, Mohyeldin A, Okagaki P, Korotchkina LG, et al. (2010) Phosphorylation status of pyruvate dehydrogenase distinguishes metabolic phenotypes of cultured rat brain astrocytes and neurons. Glia 58: 1168-1176.

6. Chesler M (2003) Regulation and modulation of pH in the brain. Physiol Rev 83: 1183-1221.

7. Siegel GJ (1994) Basic Neurochemistry: Molecular, Cellular and Medical Aspects: Raven Press, New York. 419 p.

8. Amos BJ, Chesler M (1998) Characterization of an intracellular alkaline shift in rat astrocytes triggered by metabotropic glutamate receptors. J Neurophysiol 79: 695-703.

9. Mandal A, Delamere NA, Shahidullah M (2008) Ouabain-induced stimulation of sodium-hydrogen exchange in rat optic nerve astrocytes. Am J Physiol Cell Physiol 295: C100-110.

10. McLean LA, Roscoe J, Jorgensen NK, Gorin FA, Cala PM (2000) Malignant gliomas display altered pH regulation by NHE1 compared with nontransformed astrocytes. Am J Physiol Cell Physiol 278: C676-688.

11. Svichar N, Esquenazi S, Chen H-Y, Chesler M (2011) Preemptive regulation of intracellular pH in hippocampal neurons by a dual mechanism of depolarization-induced alkalinization. J Neurosci 31: 6997-7004.

12. Zhan RZ, Fujiwara N, Yamakura T, Taga K, Fukuda S, et al. (1997) NMDA induces a biphasic change in intracellular pH in rat hippocampal slices. Brain Res 760: 179-186.

13. Xiong ZQ, Stringer JL (2000) Extracellular pH responses in CA1 and the dentate gyrus during electrical stimulation, seizure discharges, and spreading depression. J Neurophysiol 83: 3519-3524.

14. Hu Y, Wilson GS (1997) A temporary local energy pool coupled to neuronal activity: fluctuations of extracellular lactate levels in rat brain monitored with rapid-response enzyme-based sensor. J Neurochem 69: 1484-1490.

15. Deitmer JW (2002) A role for CO(2) and bicarbonate transporters in metabolic exchanges in the brain. J Neurochem 80: 721-726.

16. Azarias G, Perreten H, Lengacher S, Poburko D, Demaurex N, et al. (2011) Glutamate transport decreases mitochondrial pH and modulates oxidative metabolism in astrocytes. J Neurosci 31: 3550-3559.

17. Zhadin N, Gulotta M, Callender R (2008) Probing the role of dynamics in hydride transfer catalyzed by lactate dehydrogenase. Biophys J 95: 1974-1984.

18. DH W, Lund P, Krebs H (1967) Redox State of Free Nicotinamide-Adenine Dinucleotide in Cytoplasm and Mitochondria of Rat Liver. Biochem J 103: 514-&amp;.

19. Ido Y, Chang K, Williamson J (2004) NADH augments blood flow in physiologically activated retina and visual cortex. Proc Natl Acad Sci U S A 101: 653-658.

20. Vinnakota K, Kemp ML, Kushmerick MJ (2006) Dynamics of muscle glycogenolysis modeled with pH time course computation and pH-dependent reaction equilibria and enzyme kinetics. Biophys J 91: 1264-1287.

21. Mendes P, Kell DB, Welch GR (1995) Enzymology in Vivo; Bittar EE, editor: Elsevier Science. 1 - 19 p.

22. Nitisewojo P, Hultin HO (1976) A comparison of some kinetic properties of soluble and bound lactate dehydrogenase isoenzymes at different temperatures. Eur J Biochem 67: 87-94.

23. Gladden LB (2008) A lactatic perspective on metabolism. Med Sci Sports Exerc 40: 477-485.

24. Hashimoto T, Hussien R, Brooks GA (2006) Colocalization of MCT1, CD147, and LDH in mitochondrial inner membrane of L6 muscle cells: evidence of a mitochondrial lactate oxidation complex. Am J Physiol Endocrinol Metab 290: E1237-1244.

25. Aubert A, Costalat R, Magistretti P, Pellerin L (2005) Brain lactate kinetics: Modeling evidence for neuronal lactate uptake upon activation. Proc Natl Acad Sci U S A 102: 16448-16453.

26. Simpson IA, Carruthers A, Vannucci SJ (2007) Supply and demand in cerebral energy metabolism: the role of nutrient transporters. J Cereb Blood Flow Metab 27: 1766-1791.

27. Mangia S, Simpson IA, Vannucci SJ, Carruthers A (2009) The in vivo neuron-to-astrocyte lactate shuttle in human brain: evidence from modeling of measured lactate levels during visual stimulation. J Neurochem 109 Suppl 1: 55-62.
